# Titanium alloy composited with dual-cytokine releasing polysaccharide hydrogel to enhance osseointegration via osteogenic and macrophage polarization signaling pathways

**DOI:** 10.1093/rb/rbac003

**Published:** 2022-01-12

**Authors:** Yaping Wang, Zujian Feng, Xiang Liu, Chunfang Yang, Rui Gao, Wenshuai Liu, Wenbin Ou-Yang, Anjie Dong, Chuangnian Zhang, Pingsheng Huang, Weiwei Wang

**Affiliations:** 1 Department of Polymer Science and Engineering, Key Laboratory of Systems Bioengineering (Ministry of Education), School of Chemical Engineering and Technology, Tianjin University, Tianjin 300072, China; 2 Key Laboratory of Innovative Cardiovascular Devices, Chinese Academy of Medical Sciences, Beijing 100037, China; 3 Tianjin Key Laboratory of Biomaterial Research, Institute of Biomedical Engineering, Chinese Academy of Medical Sciences and Peking Union Medical College, Tianjin 300192, China; 4 Structural Heart Disease Center, National Center for Cardiovascular Disease, China and Fuwai Hospital, Chinese Academy of Medical Sciences and Peking Union Medical College, Beijing 100037, China

**Keywords:** hydrogel, composite scaffold, bone regeneration, cytokine delivery, macrophage polarization

## Abstract

Titanium alloy has been widely used in orthopedic surgeries as bone defect filling. However, the regeneration of high-quality new bones is limited due to the pro-inflammatory microenvironment around implants, resulting in a high occurrence rate of implant loosening or failure in osteological therapy. In this study, extracellular matrix-mimetic polysaccharide hydrogel co-delivering BMP-2 and interleukin (IL)-4 was composited with 3D printed titanium alloy to promote the osseointegration and regulate macrophage response to create a pro-healing microenvironment in bone defect. Notably, it is discovered from the bioinformatics data that IL-4 and BMP-2 could affect each other through multiple signal pathways to achieve a synergistic effect toward osteogenesis. The composite scaffold significantly promoted the osteoblast differentiation and proliferation of human bone marrow mesenchyme stem cells (hBMSCs). The repair of large-scale femur defect in rat indicated that the dual-cytokine-delivered composite scaffold could manipulate a lower inflammatory level *in situ* by polarizing macrophages to M2 phenotype, resulting in superior efficacy of mature new bone regeneration over the treatment of native titanium alloy or that with an individual cytokine. Collectively, this work highlights the importance of M2-type macrophages-enriched immune-environment in bone healing. The biomimetic hydrogel–metal implant composite is a versatile and advanced scaffold for accelerating *in vivo* bone regeneration, holding great promise in treating orthopedic diseases.

## Graphical Abstract

**Graphical Abstract:**
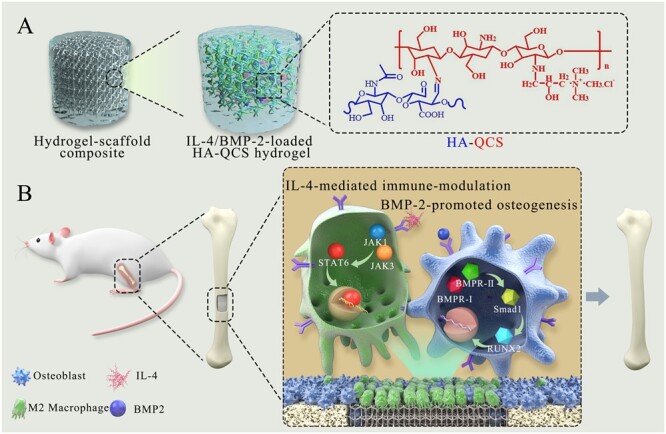

## Introduction

Bone defects caused by trauma, infection, arthritis, tumors, osteonecrosis, osteoporosis, metabolic bone disease and other diseases are widespread in clinic, causing an economic burden of over 600 000 patients every year, totaling more than 5 billion dollars in costs [1]. With the acceleration of the aging of the global population, the number of patients with bone defects is markedly increasing [2]. Repair of large bone defects has always been a great challenge in clinical treatment of orthopedic diseases. Autologous bone transplantation has been used for clinical treatment of bone defects, but the high incidence of complications caused by bone transplantation and uncontrollable number of donors limit its widespread application [3]. In addition, infection as a devastating complication frequently accompanies with the implantation of bone graft device, which requires surgical debridement and antibiotic treatment [4]. Thus, it remains highly imperative to develop alternative strategies to promote the repair and regeneration of defective bones.

Bone repair is an intricate biological process involving complex interactions between tissues, cells and cytokines, which generally comprises three phases including inflammation, repair and remodeling [5, 6]. In short, bone fractures cause the rupture of blood vessels and the formation of hematomas, leading to the recruitment of neutrophils. Meanwhile, monocytes such as macrophages would flood in the inflammatory environment to clear cell debris from dead neutrophils and apoptotic host cells. Then, mesenchymal osteochondral progenitor cells would migrate from the bone marrow to the injured site, where they proliferate and differentiate into osteoblasts or chondrocytes [7], eventually resulting in the formation of new bone. As an alternative approach to autogenous bone graft, metal scaffolds have been widely adopted as bone substitutes. For instance, three-dimensional (3D)-printed porous Ti6Al4V implants are commonly used to reconstruct bone defects due to their excellent mechanical support and osteoconductivity [8]. However, the reproduction of functional high-quality bones was far beyond the sole mechanical property, which requires the orchestration of diverse cell types and spatio-temporal regulation of different stages of bone formation. Furthermore, excessive porosity in metal scaffolds is not conducive to cell attachment, proliferation, differentiation and communication [9]. Recently, hydrogels are receiving continuous attention as implant materials for cartilage regeneration. Absorbable and bio-adhesive hydrogels could be injected or implanted locally at the defective site to facilitate the bone regeneration [10–12]. Hydrogel could also deliver pro-regenerative mineral salts including phosphate, zin ionic and silicate [13–15], to improve the osteogenic differentiation, alkaline phosphatase (ALP) activity and mineralization of human bone marrow stem cells (hBMSCs). Moreover, osteogenic cytokines, such as BMP-2, deferoxamine and stromal cell-derived factor-1 [16] or microRNA [17] that could improve the angiogenesis and osteogenesis, were also delivered in hydrogel to improve the efficacy of bone repair. However, the mechanical properties of hydrogel limit its use as a load-bearing scaffold.

**Scheme 1:**
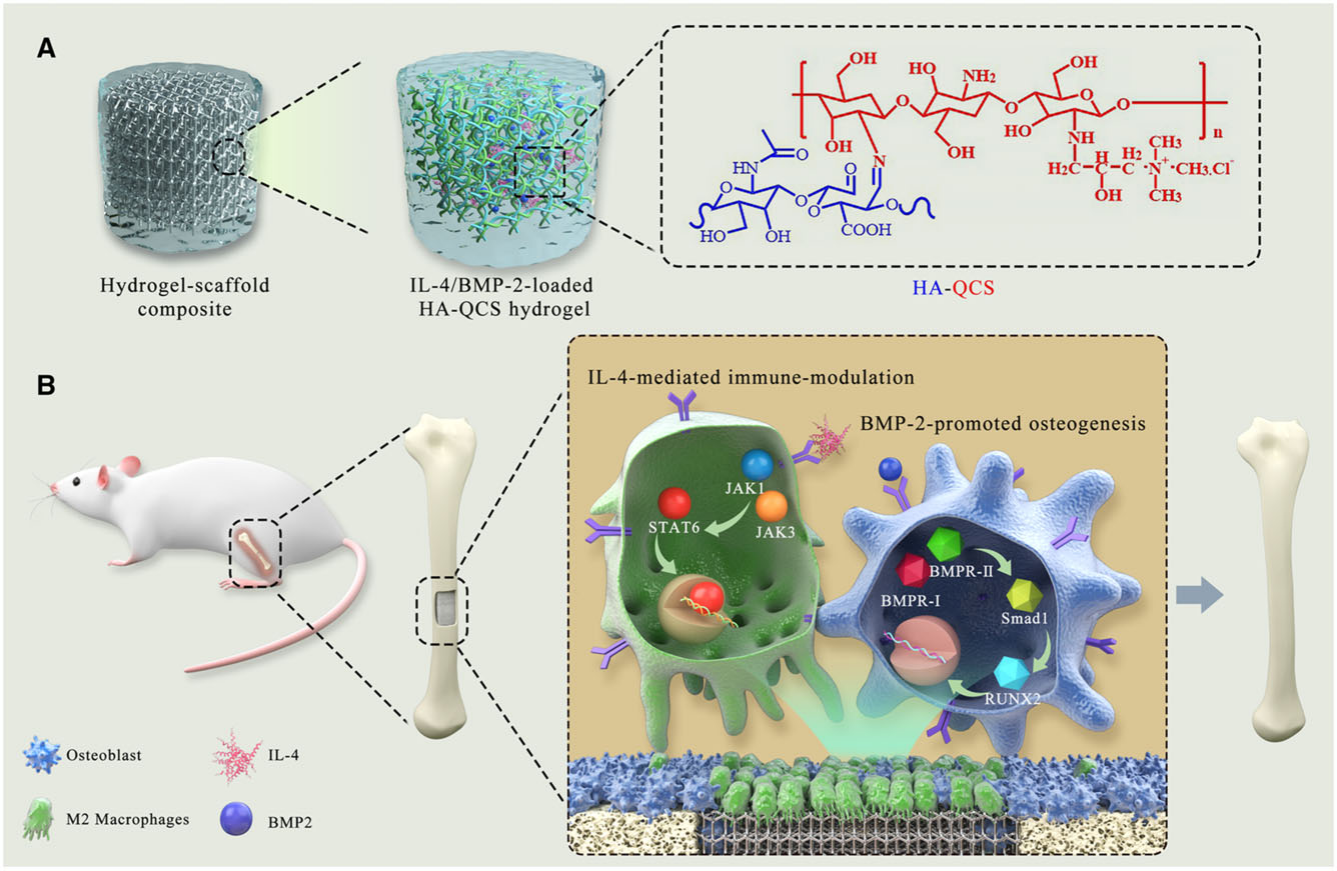
Schematic diagram of cytokine/growth factor-loaded polysaccharide hydrogel-metal scaffold composite and its role in promoting bone regeneration. ECM-inspired HQ hydrogel was complexed with Ti6Al4V scaffold, which significantly promoted in vivo regeneration of femur defect by IL-4-mediated immune-modulation and BMP-2-induced osteogenesis in the local tissue microenvironment.

After surgical implantation of scaffolds, immune cells migrate to the injured site and trigger a local inflammatory response [18], which is an indispensable step for tissue regeneration, whereas unreasonable immune response could prevent the healing process and even lead to the failure of scaffold implantation [19, 20]. The cross-talk between immune cells and other cells including osteoblasts and fibroblasts in the bone regeneration process may induce the transformation of inflammation to remodeling phase. Therefore, the immunomodulation in the damaged tissue microenvironment should be an attractive approach to improve the tissue regeneration, which remains to be elucidated. Here, biomimetic polysaccharide hydrogel–metal scaffold composite combining the merits of each composition was fabricated to promote osteogenesis for bone regeneration by modulating a pro-osteogenic immune-environment at the local bone defect site. As shown in Scheme 1, hyaluronic acid (HA), a polysaccharide widely present in the extracellular matrix (ECM) of tissues, was partially oxidized and conjugated with quaternary chitosan (QCS) through Schiff’s base reaction to obtain self-healing and porous HQ hydrogel, which could mimic the dynamic mechanical property, chemical composition and interior microstructure of natural ECM. Interleukin (IL)-4 and BMP-2 were encapsulated in the hydrogel and sustainedly delivered *in vivo* to polarize macrophages into M2-type for inducing local anti-inflammatory response, and accelerate the differentiation of osteoblasts, respectively. Then, HQ hydrogel loaded with BMP-2 and IL-4 was complexed with 3D-printed mesoporous titanium alloy scaffold to form the composite scaffold with integrated bone-induction and bone-conduction behaviors. The bioactive composite scaffold significantly improved the regeneration of femoral defects in rat by presenting a pro-regenerative immune-environment with abundant M2 macrophages. Moreover, the hydrogel showed antibacterial activity that could potentially combat the infection during scaffold implantation and bone growth. Overall, this work presented a type of versatile hydrogel–metal implant composite scaffold and provided an advanced design strategy for facilitating bone repair by combining local immunomodulation in the tissue microenvironment with osteogenesis promotion.

## Materials and methods

### Materials

Chitosan (CS) (Mw = 300 kDa; degree of deacetylation, 82%) and sodium hyaluronate (Mw = 8 kDa) were purchased from Sigma-Aldrich. Information for other chemical and biological agents were provided in the supporting information. Sprague Dawley (SD) rats (12 weeks, 300 g) and female BALB/c (6–8 weeks) mice were purchased from SIPEIFU (Beijing, China). All animal procedures were performed abide by the rules established by Authority of Institute of Radiation Medicine, Chinese Academy of Medical Sciences (Approval No: SYXK (Jin) 2019-0002) and Animal Experiment Ethics Committee.

### Methods

#### Preparation and characterization of Ti alloy–hydrogel composite scaffold

##### Design and fabrication of the 3D Ti6Al4V implant

A cylindrical porous Ti6Al4V implant with a mesh size of 2 mm, a porosity of 55% and a pore size of 300 µm was fabricated by 3D printing. The porous Ti6Al4V alloy was machined into scaffolds with a diameter of 2.5 mm and a length of 3 mm, namely Ti (D = 2.5 mm, L = 3 mm). Spherical pure titanium powder Ti6Al4V was used as the ink and scaffold graphics were designed by CAD software (Magics, Materialise, Belgium); 3D printing was performed by the ProX DMP 320 machine (3D Systems, Leuven, Belgium). The printed scaffold was cleaned by ultrasonic in demineralized water to remove excess particles and pollutants.

##### Synthesis of QCS and Hyaluronic acid bearing aldehyde groups

For the synthesis of QCS, CS (5 g) was dissolved in 1% acetic acid solution by stirring at room temperature for 30 min, and then glycidyl trimethylammonium chloride (GTMAC, 14 g) was added, and the mixture was stirred at 55°C for 18 h. The solution was dialyzed in deionized water for 3 days and then lyophilized to obtain QCS as white solids. Hyaluronic acid bearing aldehyde groups (HA-CHO) was synthesized by oxidation reaction. Briefly, HA (4 g) was dissolved in distilled water (600 mL), and then sodium periodate (0.8 g) in aqueous solution was slowly added. The reaction was continued at room temperature for 2 h in dark, and terminated by adding excess ethylene glycol, which was dialyzed in water for 5 days. HA-CHO was obtained by freeze-drying and stored under nitrogen atmosphere.

##### Preparation of hydrogel

HQ hydrogel was prepared by Schiff’s base reaction between aldehyde groups in HA and amino groups in CS. Briefly, an appropriate amount of HA-CHO or QCS were dissolved in deionized water and stirred until completely dissolved. Subsequently, the two solutions were mixed to form the hybrid HQ. A series of hydrogels were prepared by adjusting the initial feeding mass ratio of substances or the total polysaccharide concentration. To prepare cytokine-encapsulated hydrogel, BMP-2 (100 ng) and IL-4 (100 ng) were directly mixed in the phosphate buffer solution (PBS, 100 µL) of HA-CHO (3 µg) or QCS (3 µg). HQ (200 µL) encapsulated with cytokines were named as BMP2-HQ (100 ng, 200 µL), IL4-HQ (100 ng, 200 µL) and BMP2/IL4-HQ (100 ng, 100 ng, 200 µL), respectively.

##### Preparation of Ti alloy–hydrogel composite scaffold

To prepare the composite scaffold of 3D printed titanium alloy and HA/CS hybrid hydrogels, BMP-2 (100 ng) and IL-4 (100 ng) were directly dissolved in the PBS (100 µL) of HA-CHO (3 µg) or QCS (3 µg). The mixture was processed by vortex for 5 s, which was cultured with titanium alloy in the mold for several minutes to allow the formation of Ti alloy–hydrogel composite scaffold.

The characterization of polymers, hydrogels and composite scaffolds including FT-IR spectrum, rheology, SEM examination, degradation and cytokine release was shown in the supporting information.

#### Public database analysis

We searched ‘bone defect’ in the Gene Expression Omnibus (GEO) database. Microarray datasets (GSE106325, platform: GPL570 Platform, [HG-U133_Plus_2] Affymetrix Human Genome U133 Plus 2.0 Array) were downloaded, which enrolled human bone marrow-derived mesenchymal stem cells (hBMSCs) either cultured alone or co-cultured with human colon cancer cells HT29 or HCT116, respectively. Differentially expressed genes (DEGs) were filtrated by GEO2R. R language software (version 4.0.0) was used to construct volcano maps. Then, the database DAVID (version 6.8) was used to perform functional and pathway enrichment analysis on the selected DEGs, and results were visualized by R software. Finally, the protein–protein interaction (PPI) network of mitogen-activated protein kinase (MAPK) signaling pathway was constructed and adjusted using the database of Search Tool for the Retrieval of Interaction Gene/Proteins (version 11.0) and Cytoscape software (version 3.7.2).

#### Assessment of bone repair in vitro

##### Cell culture

Human bone marrow stem cells (hBMSCs) were cultured with DMEM medium (10% fetal bovine serum; 100 units/mL penicillin; 100 units/mL streptomycin) and incubated (37°C, 5% CO_2_). Prior to cell culture, HA-CHO or QCS was dissolved in PBS at a concentration of 3 wt%, which was filtered by a 0.22 µm filter. The 3D-printed Ti6Al4V scaffolds were immersed in alcohol and sterilized by ultraviolet radiation for half an hour. The scaffold was placed in the plate and HQ was prepared on-site by adding the mixture of HA-CHO and QCS solution to allow gelation. After trypsin digestion, hBMSCs were seeded in a 24-well plate (1 × 10^5^ cells/well). The absorbance at 450 nm was measured with a microplate reader (Thermo Scientific Appliskan, USA). All experiments were independently repeated for three times.

##### Proliferation, adhesion and differentiation of hBMSCs

For cell adhesion assay, hBMSCs cells were seeded in 24-well plates (1 × 10^5^ cells per well) for 5 or 24 h, respectively. Afterwards, the original culture medium was removed by pipette tips and samples were rinsed twice with sterile PBS. Cells were seeded on the scaffold as described above and then fixed with 4% polyoxymethylene, and the nuclei and cytoskeleton protein F-actin were stained with 4′,6-diamidino-2-phenylindole (DAPI) and Actin-Tracker Red-555, respectively. To study the effect of BMP2-HQ on the differentiation of hBMSCs, the expression of ALP was determined at Day 3, 7 and 14. ECM mineralization and collagen secretion were also examined at Day 21. Details for these experiments were supplemented in the supporting information file.

#### Polarization of macrophages in vitro and in vivo

Bone marrow-derived macrophages (BMDMs) were extracted and isolated from BALB/c mice according to the previous report [21]. BMDMs were seeded in a confocal culture dish and incubated with Titanium scaffold (Ti, D = 2.5 mm, L = 3 mm), Ti@HQ, Ti@BMP2-HQ, Ti@IL4-HQ and Ti@BMP2/IL4-HQ for 48 h. The dose for each cytokine was 100 ng and the volume for hydrogel was 200 µL. To identify the polarization state of macrophages, Actin-Tracker Red-555 and DAPI were used to label cytoskeleton and cell nuclei, respectively. And M2-type macrophages were identified by the surface typical biomarker CD206, confocal laser scanning microscopy (CLSM) was used to capture pictures (TCS SP5II, Leica, Ernst-Leitz-Strasse, Germany).

BMDMs were seeded in 6-well plates (4 × 10^5^ cells per well) and treated as described above. Cells were collected by centrifugation and stained with antibodies against CD68, F4/80 and CD206. CD68 is a 110 kD glycoprotein, belonging to the sialomucin family, and predominately expressed in cytoplasmic granules of monocytes/macrophages, which could be used as a typical marker of M1-type macrophages. F4/80 is a 160 kD glycoprotein and a member of the epidermal growth factor-transmembrane 7 family, which has been widely used as a murine macrophage marker. CD206, also known as mannose receptor, is a 175 kD Type I membrane protein and a pattern recognition receptor belonging to the C-type lectin superfamily, which is a typical marker of M2-type macrophages.

For *in vivo* macrophage polarization, SD rats (300 g, 12 weeks old; *n* = 5 per group) were anesthetized by inhalation of isoflurane, and circular holes with a diameter of 2.5 mm and a depth of 3.3 mm were made with a bone drill. The prepared composite scaffold was implanted at the defect location. The wound was carefully sutured and disinfected. Four weeks later, muscle and new bone tissues at the implantation site were separated and sliced. Then, the sections were stained with the primary antibody of runt-related transcription factor 2 (RUNX2), inducible nitric oxide synthase (iNOS), CD68 and CD206. After standing in the incubator for 3 h, the tissue sections were transferred to PBS for washing, and goat anti-rabbit IgG and goat anti-mouse IgG were sucked onto the tissue sections. Finally, the sections were stained with DAPI, and images were captured by CLSM and quantitatively analyzed by Image J software.

#### Real-time quantitative reverse transcription polymerase chain reaction and western blot

BMDMs were seeded in a confocal culture dish and treated with various scaffolds for 48 h. Then, the culture medium was collected and centrifuged to obtain the supernatant. Total RNA was isolated and extracted from BMDMs, and transcribed using GoScript reverse transcription system (Promega, A5001). Real-time PCR was performed using SYBR Green Master Mix (Promega, A6001) to test the gene expression for M1-related cytokines including interferon regulatory factor 5 (IRF5) and IL-6, and M2-related cytokines including signal transducers and activators of tranion6 (STAT6) and IL-10. Details for PCR and western-blot assays were provided in the supporting information.

#### Assessment of bone repair in vivo

##### Implantation of hydrogel–scaffold composite scaffold in femoral defects

SD rats (300 g; *n* = 8 for each group) were anesthetized by inhalation of isoflurane. The femur of each rat was fixed, and the legs were depilated. A circular hole with a size of 2.5 mm in diameter and 3.3 mm in depth was made by a bone drill. The 3D-printed scaffold mixed with hydrogel was implanted in the defect and the wound was sutured and disinfected carefully. In order to avoid the adverse influence of titanium alloy on Micro-CT imaging and histopathological analysis, we used hollow cylindrical titanium alloy (2.5 mm in diameter and 3.3 mm in depth) in the femoral defect model to replace the 3D printed titanium alloy scaffold. The femur was harvested and fixed in 4% (w/v) paraformaldehyde for 2 days, mounted in a poly (methyl methacrylate) holder, and subjected to Micro-CT analysis to evaluate the bone repair.

##### Micro-CT scanning and data analysis

To evaluate bone defect repair at the time points of interest (Weeks 4 and 8), micro-computed tomography (Micro-CT, SkyScan 1176, Bruker, Belgium) imaging was performed. Animals were anesthetized by isoflurane inhalation, and the femurs were collected and fixed in 4% (w/v) paraformaldehyde. The samples of bone tissues were scanned by a Micro-CT at 45 kV, 177 μA, and the X-ray effective energy was about 24 keV, integration time of 200 ms and resolution of 15 microns. The contour of the inner radius of the implant on each two-dimensional section was draw, and then Gaussian filter was applied to quantify the bone volume. After 3D reconstruction, the bone mineral density (BMD) and the volume ratio of bone to tissue area (BV/TV) were determined.

##### Hematoxylin and eosin, Masson’s and immunohistochemistry staining

Hematoxylin and eosin (H&E) and Masson’s trichrome staining were performed to determine the extent of new bone formation. After decalcification, tissue samples were embedded in paraffin and sectioned longitudinally. Then the sections were stained with H&E and Masson’s trichrome to identify the characteristic cells and tissues during bone regeneration. Immunohistochemistry was carried out using a two-step detection kit. Experimental details were described in the supporting information. Images were captured by CLSM and quantitatively analyzed by Image J software.

#### Antibacterial test

Gram-positive and Gram-negative bacteria strain, including *Staphylococcusaureus* and *Escherichiacoli* were used to measure the antibacterial ability of HQ *in vitro* and *in vivo*. Experimental details were described in the supporting information.

### Statistical analysis

All experiments were repeated three times at least, and results were shown as mean ± standard deviations (SDs). The statistical difference between two groups or multiple groups were analyzed using Student’s *t*-test or one-way ANOVA, respectively. Statistical significance is denoted by *P < 0.05, **P < 0.01 and ***P < 0.001.

## Results

### Preparation and characterization of HQ hydrogel

ECM is a highly complex and porous network of various bioactive substances [4], including diverse polysaccharides and proteins, which plays a crucial role in cell proliferation and tissue development. Hydrogels with high water content and 3D crosslinked porous structures could mimic the essential architecture of ECM and serve as delivery vehicles for growth factors [22], to expedite tissue regeneration. The adaptable hydrogels simulating the reversible connection of native ECM can provide mechanical support for cells [23]. Hydrogels with irreversible bonds do not have the properties of self-healing, shear-thinning and fast stress-relaxation properties [24]. Thus, biodegradable dynamic polymers attract numerous attentions, which could endow dynamic changes in cell proliferation and tissue regeneration [25, 26]. The latest developments in dynamic chemistry have produced multiple designs of adaptive hydrogels [27]. Dynamic covalent bonds, including hydrazine bonds, imine bonds, hydrazine bonds and borate esters, can be broken and reformed under equilibrium conditions. Of these covalent bonds, imine bonds obtained through the Schiff base reaction such as aldehyde-amines and ketone-amines, possesses mild reaction conditions, high yield efficiency, separation and recombination with external stimuli. In addition, a wide range of substances such as proteins and polysaccharide have amine groups, enabling imine bonds an ideal candidate for fabricating adaptive hydrogel. Herein, inspired by the chemical component, structure and function of ECM, HA grafted with QCS was synthesized to prepare a two-component hydrogel that could ideally mimic the polysaccharide component in natural ECM, and other specific characters of ECM including water-rich environment, highly porous network structure and dynamic adaptability.

**Figure 1.**
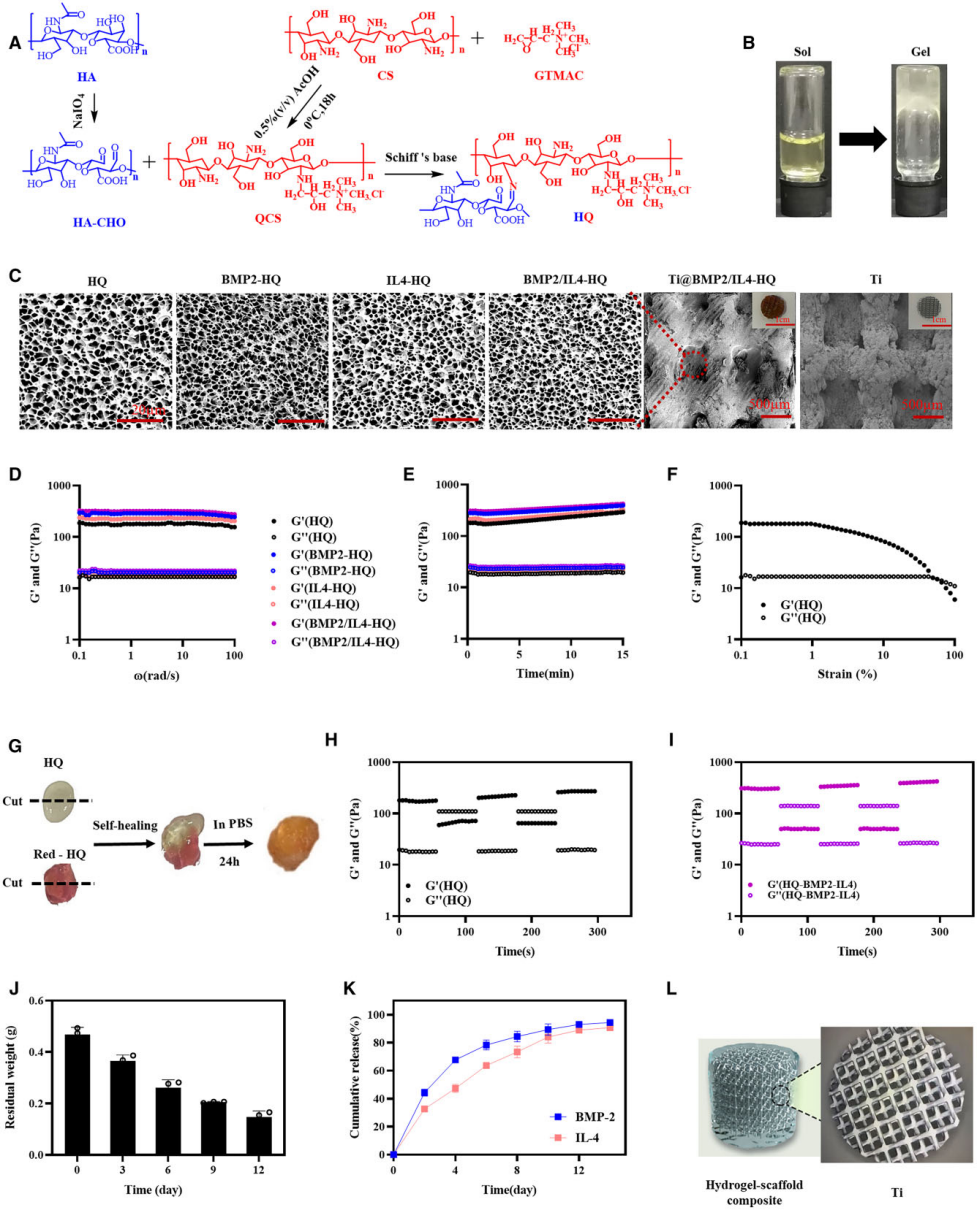
Preparation and characterization of ECM-inspired, self-healable and biodegradable HQ hybrid hydrogel. (A) The synthesis route of HA-CHO, QCS and HQ. (B) The sol–gel transition of HQ. (C) Representative SEM images of lyophilized hydrogel. (D and E) The rheological analysis of hydrogel with angular frequency (D) and time (E) sweep. (F) The change in modulus of HQ hydrogel as a function of shear strain. (G–I) Self-healing analysis of HQ hydrogel. (J) The degradation of HQ hydrogel at 37°C in the presence of hyaluronidase. (K) The cumulative release profile of BMP-2 and IL-4 from the hydrogel *in vitro*. (L) The hydrogel–scaffold composite

First, HA was oxidized to generate aldehydes and hemiacetals and the product was denoted as HA-CHO (Fig. 1A), which could react with amines to form imine bonds. The degree of oxidation was ∼ 41.37% as determined by colorimetric hydroxylamine titration and ^1^H NMR (Figs S1and S2). Then, quaternization of CS was performed by GTMAC via amine-initiated ring-opening reaction. The characteristic chemical shift at 3.1 ppm attributed to –CH_3_ groups in the quaternary ammonium salt indicated the successful synthesis of quaternized chitosan (QCS, Fig. S3). The molar ratio of quaternary ammonium groups in QCS was about 30%. Afterward, the solutions of HA-CHO and QCS with a concentration of 3% (w/v) were mixed at a volume ratio of 1:2, 1:1 and 2:1, respectively. HQ hydrogels were spontaneously formed through Schiff’s base reaction between amine and aldehyde groups (Figs 1A and S4), despite the different volume ratios between HA-CHO and QCS solution. The HQ hydrogel produced at the volume ratio of 1:1 with porous structure and moderate elastic modulus that could mimic the architecture and mechanical property of natural ECM was used as a representative specimen for following proof-of-concept studies. All these polysaccharide derivatives and composite polymers were further characterized by FT-IR spectrum (Fig. S5). Characteristic stretching vibration peak for –NH– at 1590 cm^−1^ and for –CH_3_ in the quaternary ammonium group of QCS at 1486 and 1653 cm^−1^ appeared. FT-IR spectrum of HQ shows the characteristic deformation vibration peak assigned to the imine bond at 1631 cm^−1^, indicating the chemical coupling between HA-CHO and QCS. In addition, IL-4 and BMP-2 could be easily encapsulated into HQ hydrogel (named as BMP2/IL4-HQ) by dissolving an individual or dual macromolecules in the precursor solution to construct the delivery system.

Subsequently, sol-to-gel phase transformation of HQ is shown in Fig. 1B. Internal morphology of the hydrogel observed by SEM showed that all hydrogel samples exhibited a highly porous microstructure with a uniform and tight network (Fig. 1C) and the hydrogel was homogeneously distributed in the Ti alloy scaffold. It is expected that the hydrogel with porous structure and higher water content could support the cell inlay and facilitate the transfer of nutrients and cytokines between cells. Rheology analysis (Fig. 1D and E) demonstrated that the storage modulus (G') of HQ hydrogel was higher than the loss modulus (G''), within the angular frequency of 0.1–100 rad/s or the incubation time of 0–15 min, indicating the formation of stable viscoelastic hydrogel. The introduction of BMP-2 and IL-4 did not significantly influence the modulus of HQ hydrogel. Additionally, HQ hydrogel could be easily injected through a syringe equipped with a 25G needle (Fig. S6a), reflecting the injectability of HQ hydrogel. Figure 1F showed that HQ hydrogel displayed the shear-thinning property (non-Newtonian). With the shear strain increased, the G' and G'' have a decreasing trend and decrease in G' more significant. At 56.9%, the G'' and G' line intersects. After the intersection, G'' > G', which further proves the shear thinning of the HQ hydrogel. The dynamic imide linkages and hydrogen bonds present in HQ may render HQ hydrogel self-healing ability. Native HQ and rhodamine B (RB)-labeled HQ hydrogels were cut into two pieces (Fig. 1G), which were held together along the cut surface without any external forces. It was found that the two pieces could spontaneously merge together and the boundary was completely blended after being immersed in PBS for 24 h. The modulus of HQ or BMP2/IL4-HQ hydrogel was decreased upon applying a large stain of 50%, and quickly recovered to the gel state at a small strain of 2% (Fig. 1H and I). These data suggested that HQ was injectable, self-healing and dynamic-adaptive. Moreover, the swelling ratio (Fig. S6b) of HQ hydrogels was around 25-fold of the initial mass.

Then, the degradation and drug release behavior for HQ hydrogel were assessed. Since Ti6Al4V implants are non-degradable due to corrosion resistance and persistently present in the human body [28], the degradation of HQ hydrogel alone was determined. Figure 1J showed that HQ hydrogel displayed a time-dependent degradation profile. At Day 12, the degradation rate was about 80%. Fluorescence imaging was performed to observe the hydrogel retention *in vivo* after subcutaneous injection of RB-labeled HQ hydrogel. The fluorescence signal of HQ hydrogel was gradually decayed over time and a linear relationship between total fluorescence signal and time was determined (Fig. S7). Almost no fluorescence signal was detected at Day 14 post injection, indicating the complete degradation of hydrogel. *In vitro* cytokine release (Fig. 1K) indicated that BMP-2 and IL-4 could be sustainedly released from BMP2/IL4-HQ hydrogel without obvious burst release. The accumulative release for BMP-2 and IL-4 was ∼ 95% and 90%, respectively, after 14 days. Because BMP-2 and IL-4 were physically entrapped in the hydrogel, they would diffuse out of the hydrogel as driven by the concentration gradient when hydrogel matrices were exposed to the aqueous environment. Meanwhile, HQ was degradable, which would also result in the release of BMP-2 and IL-4. Hence, it was suggested that the release of cytokines from HQ hydrogel was dominated by both drug diffusion and hydrogel degradation. These results demonstrated that biodegradable HQ hydrogel could serve as a controlled delivery platform for BMP-2 and IL-4. In this study, the precursor HA-CHO and QCS solutions were mixed with the Titanium scaffold *in vitro* to prepare the composite scaffold (Ti@HQ, Fig. 1L), which was anticipated to confer the scaffold better osteoinduction capability and immunomodulation ability, which might improve the bone repair. In brief, to prepare the composite scaffold of 3D printed titanium alloy and HA/CS hybrid hydrogels, BMP-2 (100 ng) and IL-4 (100 ng) were directly mixed in the PBS (100 µL) of HA-CHO (3 µg) or QCS (3 µg). Next, the porous Ti6Al4V alloy (D = 2.5 mm, L = 3 mm) was mixed with the precursor HA-CHO (3 µg) and QCS (3 µg) solutions (PBS, 100 µL) including BMP-2 (100 ng) and IL-4 (100 ng). The composite scaffold occurred in 1–5 s upon vortex.

### Cytocompatibility and osteoinductivity of cytokine-delivered HQ hydrogel

**Figure 2.**
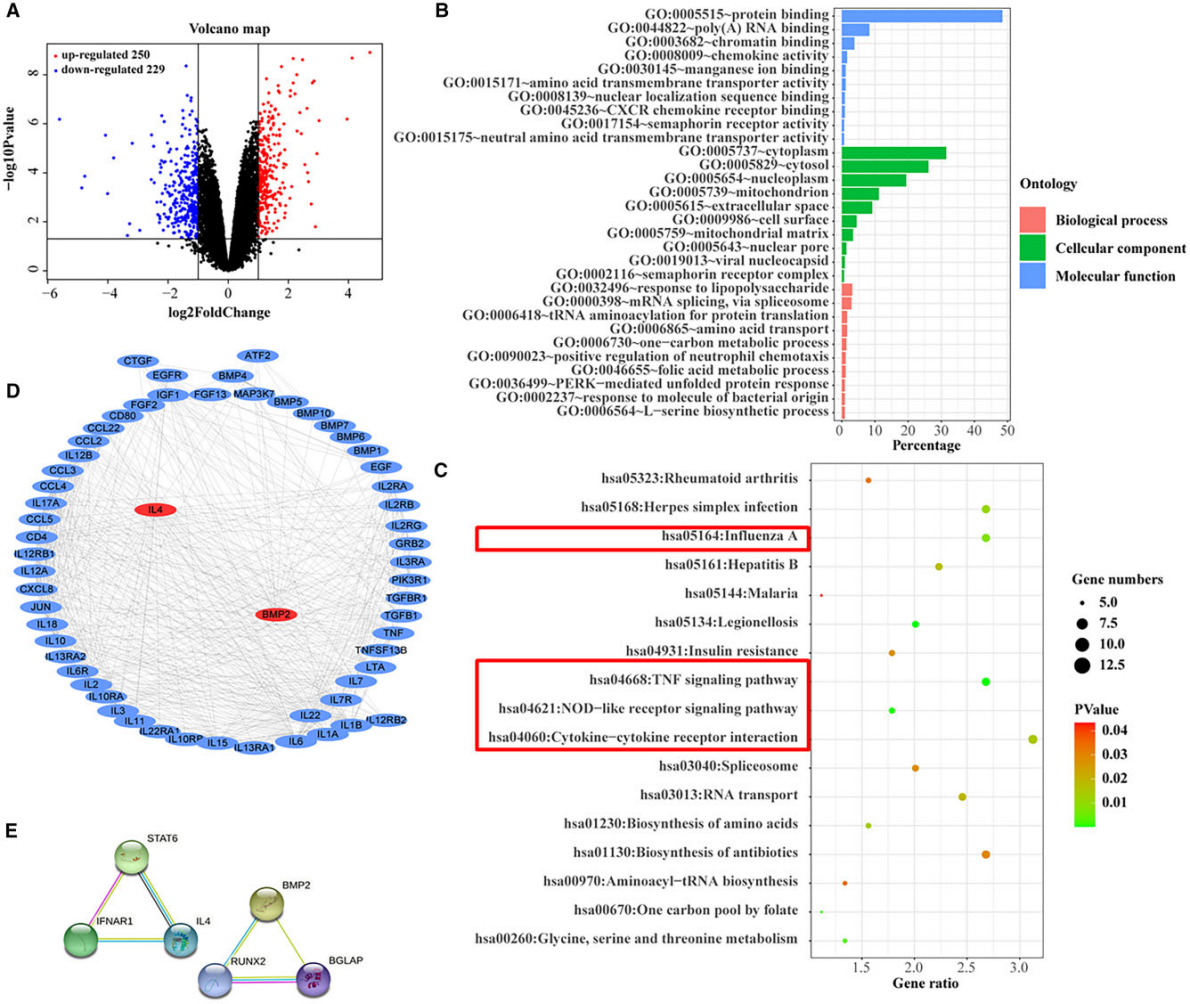
Results of transcriptome sequencing and pathway analysis. (A) Volcano plot visually showing the number of up-regulated (red dots) and down-regulated genes (blue dots). Cut-off: corrected *P* values < 0.05 and |log FC| > 1. (B) GO analysis indicating the biological functions of differentially expressed genes, including biological process (BP), cellular component (CC) and molecular function (MF). (C) KEGG pathway analysis demonstrating the top 17 signal pathways enriched by differentially expressed genes. (D) Protein–protein interaction (PPI) network involved in osteogenesis-related signaling pathways generated by Ingenuity Pathway Analysis software. (E) Independent signaling pathways of IL-4 and BMP-2

Bioinformatics analysis was performed to reveal the synergetic mechanism of osteogenic differentiation induced by IL-4 and BMP-2. A total of 479 DEGs were selected from GSE106325 according to the criteria (corrected *P* values < 0.05 and |log FC| >1), including 250 and 229 genes with up-regulated and down-regulated expression, respectively, which were closely related to bone formation and angiogenesis. The volcano map is shown in Fig. 2A. The Gene Ontology (GO) and key pathways were obtained during functional enrichment analysis by using DAVID database. The significant GO enrichment results are shown in Fig. 2B. DEGs were mostly involved in protein binding, RNA binding, chromatin binding, chemokine activity, ion binding, channel activity, metal ion binding, transmembrane transporter activity, etc.; 17 key pathways were obtained from functional enrichment analysis according to DEGs (Fig. 2C). IL-4 and BMP-2 participated in many enriched pathways. IL-4 and BMP-2 could influence each other through multiple signal pathways such as MAPK belonging to non-obese diabetic-like receptor signaling pathway, TNF signaling pathway and Influenza A, which are marked in red box in Figs 2C and S8. Then the PPI network of genes in MAPK signaling pathway was built, and the interaction between IL-4 and BMP-2 is displayed in Fig. 2D, which vividly showed the molecular interaction network of DEGs involved in osteogenesis-related signaling pathways. As observed in the independent signaling pathways of IL-4 and BMP-2 in Fig. 2E, we could know that IL-4 and BMP-2 interacts with STAT6 and RUNX2, respectively. Based on the comprehensive analysis of transcriptome sequencing and PPI results, IL-4 and BMP-2 could affect each other under the MAPK pathway to achieve a synergistic effect toward osteogenesis. Thus, BMP-2 and IL-4 were used for the following osteoinductivity study.

3D-printed porous Ti6Al4V implants are usually used to reconstruct bone defects due to their excellent mechanical support and osteoconductivity. However, the osteoinduction of these implants needs to be further improved [29]. The HQ hydrogel with ECM-like components and porous 3D network structure, self-healing properties and controlled cargo release can not only provide a suitable microenvironment for cell infiltration and proliferation, but also may facilitate osteoinduction. In this study, the precursor HA-CHO and QCS solutions with or without the growth factor and cytokine were mixed with the Titanium scaffold *in vitro* to prepare the composite scaffold (Ti@HQ, Scheme 1A), which was anticipated to confer the scaffold better osteoinduction capability and immunomodulation ability that might improve the bone repair.

**Figure 3.**
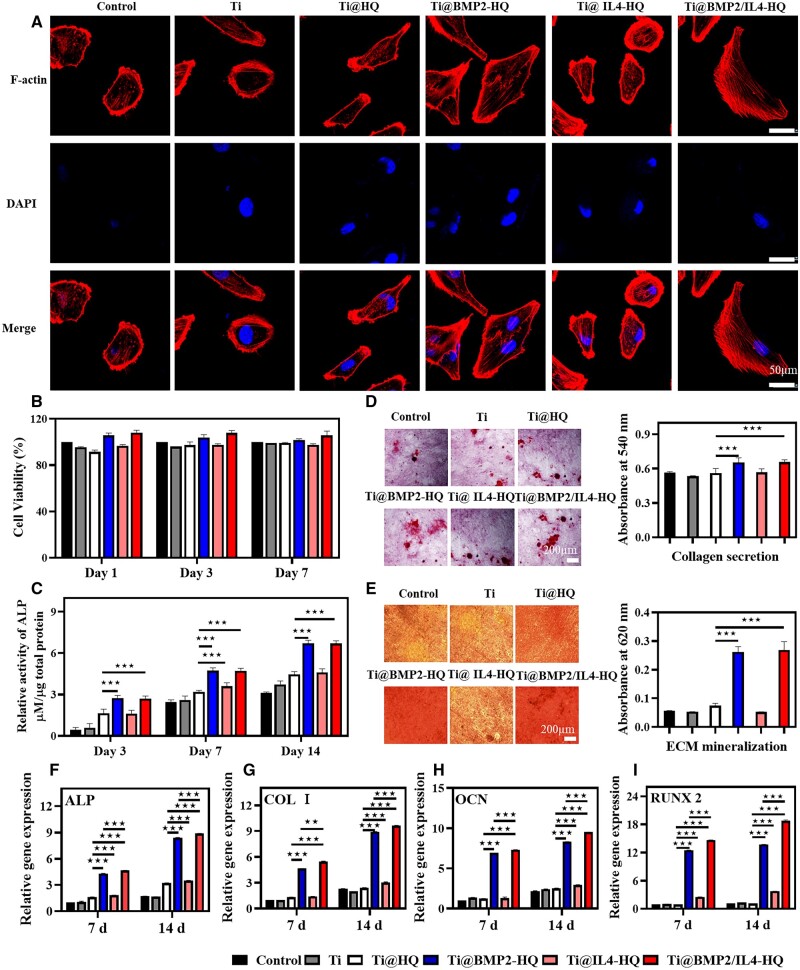
The osteogenic differentiation of hBMSCs. (A) *In vitro* ages of hBMSCs cultured on the scaffold for 5 h. Red channel, F-actin; blue channel, cell nuclear. (B) The viability of hBMSCs cultured for 1, 3 and 7 days. (C) The ALP activity of osteoblasts after culturing hBMSCs for 3, 7 and 14 days. The content of ALP was normalized to the total protein. (D and E) Collagen secretion (D) and ECM mineralization (E) of hBMSCs after incubation for 21 days. (F–I) The expression of osteogenesis-associated genes for ALP (F), COL I (G), OCN (H) and RUNX2 (I). *P < 0.05, **P < 0.01 and ***P < 0.001, between indicated two groups, Student’s *t*-test

The fracture area begins to heal at the beginning of inflammation. Several biological factors including transforming growth factor-beta and BMP superfamily are released. BMP-2 plays a vital role in bone healing process [30], acting as a high-efficiency osteogenic factor [31]. BMP-2 has been approved by the US Food and Drug Administration for clinical orthopedics [32, 33]. Besides the immunomodulating property, IL-4 was also found to influence both osteoclasts and osteoblasts. It has been identified that IL-4 could directly prevent the formation and function of osteoclasts via STAT6 and peroxisome proliferators-activated receptor γ-mediated inhibition of nuclear factor kappa B activation, which could benefit the improvement of osteoblast activity *in vivo* [34]. Here, BMP-2 and IL-4 were encapsulated in HQ to accelerate the differentiation of osteoblasts. First, the cytocompatibility of HQ was evaluated. hBMSCs were cultured with various scaffolds and stained by live/dead kit. Compared with native titanium alloy scaffold, the incorporation of HQ hydrogel with or without BMP-2 increased the number of living cells, which was evidenced by a larger number of green fluorescent spots (Fig. S9). Furthermore, as shown in Fig. 3A, the morphology of cells cultured on titanium alloy for 5 h were small and narrow in shape with a few pseudopodia. However, cells cultured in Ti@HQ, Ti@BMP2-HQ and Ti@BMP2/IL4-HQ were larger in diameter (Fig. S10), and polygonal or spindle-shaped with evident filose pseudopodium and flat membranes. After further incubation for 24 h, hBMSCs with pseudopod-like structures proliferated rapidly (Fig. S11). The viability of hBMSCs on Ti@BMP2/IL4-HQ was higher than 100% at scheduled time points (Fig. 3B), demonstrating that Ti@BMP2/IL4-HQ could enhance the proliferation and osteogenic differentiation of hBMSCs. Moreover, the hemolysis ratio for HQ and BMP2/IL4-HQ hydrogel was < 5% (Fig. S12), suggesting HQ hydrogel was blood-compatible.

Then, the osteoinductive activity of composite scaffold was evaluated. As shown in Fig. 3C and D, the ALP expression and collagen dispersion of hBMSCs treated by Ti@BMP2-HQ or Ti@BMP2/IL4-HQ was conspicuously increased in comparison with Ti@HQ and Ti@IL4-HQ. Alizarin Red S was used to stain calcium to detect ECM mineralization. As shown in Fig. 3E, ECM mineralization induced by Ti@BMP2/IL4-HQ and Ti@BMP2/IL4-HQ is about 5-fold of that in other groups. These colorimetric analyses proved that BMP-2 delivered by HQ hydrogel can promote the osteogenic differentiation of hBMSCs. In addition, real-time quantitative reverse transcription polymerase chain reaction was used to quantitatively analyze the gene expression of typical bone-formation markers including collagen I (COL I), ALP, RUNX2 and osteocalcin (OCN) (Table S1). As shown in Fig. 3F–I, compared with Ti or Ti@HQ, these osteogenesis-related genes were pronouncedly up-regulated in both Ti@BMP2-HQ and Ti@BMP2/IL4-HQ groups. Specifically, on Day 7, the ALP expression of the Ti@BMP2-HQ and Ti@BMP2/IL4-HQ were 2.6-fold and 2.8-fold of that in Ti@HQ group, respectively. The ALP expression of cells treated by Ti@BMP2-HQ and Ti@BMP2/IL4-HQ was markedly increased when the culturing time period was prolonged to 14 days, in comparison with that obtained at Day 7. Moreover, Ti@BMP2/IL4-HQ showed significantly higher efficacy in promoting the expression of osteogenesis-related genes encoding proteins including COL I, ALP, RUNX2 and OCN, in comparison with Ti@BMP2/-HQ. IL-4 treatment can also promote the expression of osteogenic genes, which is conducive to improve the osteoblast differentiation. These data suggested that BMP-2 and IL-4 delivered in HQ could promote the adhesion and proliferation, the collagen secretion, ECM mineralization and the expression of genes related with the osteogenic differentiation of hBMSCs. Thus, the construction of Ti@BMP2/IL4-HQ composite scaffold provided a feasible approach for facilitating *in vitro* osteogenic differentiation of hBMSCs.

### Macrophage polarization *in vitro* and *in vivo*

**Figure 4.**
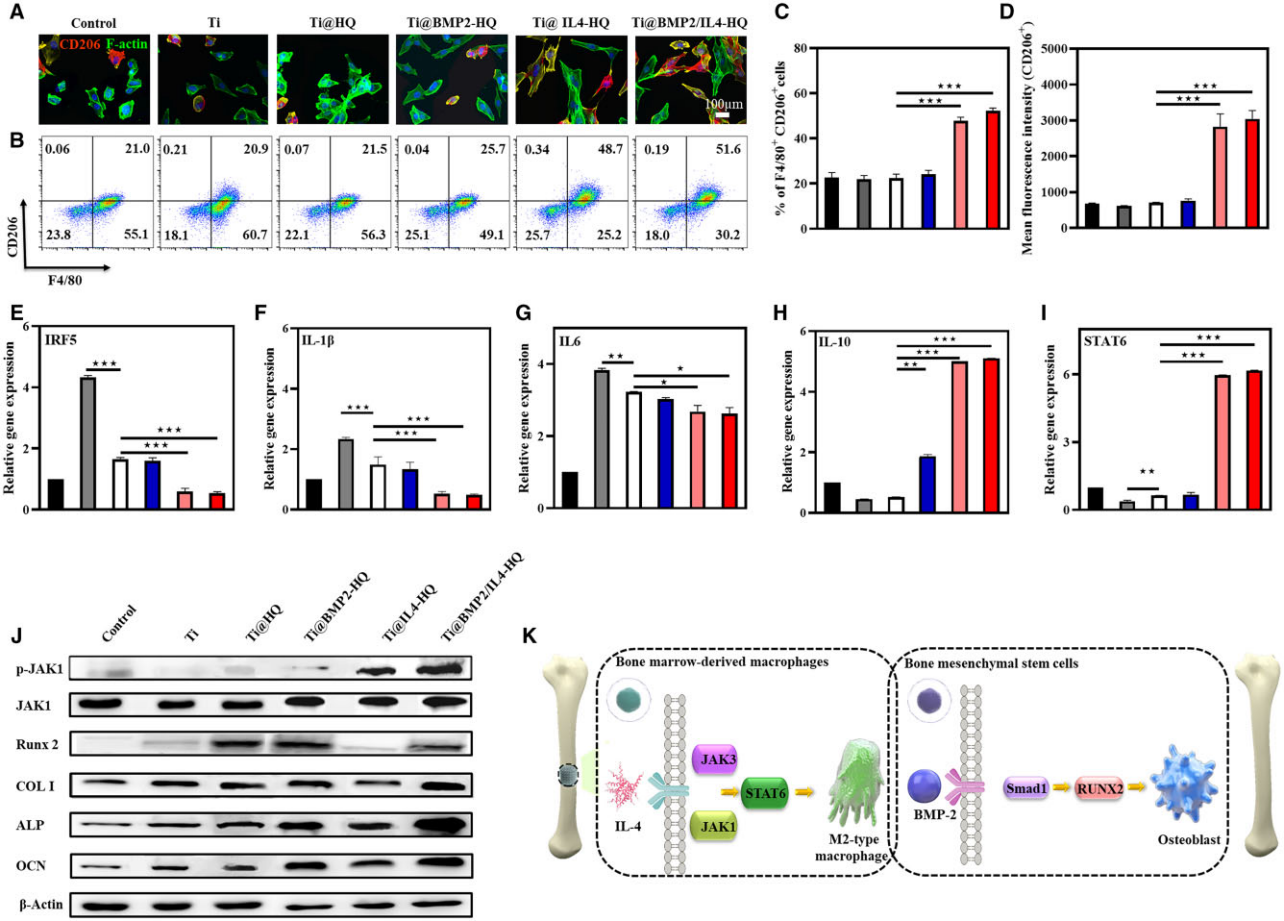
Ti@HQ scaffold loaded with IL-4 effectively primed macrophages toward M2 phenotype. (A) Representative CLSM images of BMDMs treated for 48 h. Red channel, CD206; green channel, F-actin; blue channel, nucleus (*n* = 3). (B) Flow cytometry analysis of CD206 expression and the percentage of M2 type (C, F4/80^+^CD206^+^) macrophages. The gating approach for flow cytometry was based on FSC-A and SSC-A (Fig. S13) (*n* = 3). (D) Mean fluorescence intensity of CD206 staining (*n* = 3). (E–I) Real-time PCR of M1 polarization-related IRF5 (E), IL-1β (F) and IL-6 (G) and M2 polarization-related IL-10 (H) and STAT6 (I) (*n* = 3). (J) Protein expression level of p-JAK1, and JAK1 in macrophages runx-2, COL I, ALP and OCN in hBMSCs determined by western blotting. (K) The mechanism of M2-type macrophage polarization and osteogenic gene activation by Ti@BMP2/IL4-HQ. Data are shown as mean ± SDs (*n* = 5). **P < 0.01, ***P < 0.001, between the indicated groups

The phenotype of macrophages is closely related to the inflammation during tissue regeneration, which is typically divided into pro-inflammatory M1-type and pro-regenerative M2-type [35]. Particularly, IL4 or IL13 is commonly used to induce macrophage M2-type polarization [36]. However, the use of high-dose ILs could consequentially result in off-target side effects [37]. The controlled release of IL-4 by HQ hydrogel may reduce side effects while prime the naïve macrophages into M2 phenotype. CD206 was selected as the surface marker for M2-type macrophages. Figure 4A depicts the morphology of BMDMs treated for 48 h. In contrast to the control group, BMDMs incubated with Ti@IL4-HQ and Ti@BMP2/IL4-HQ both showed obvious pseudopod-like structure and stronger red fluorescence intensity of CD206 staining, indicating that macrophages were polarized into M2-type phenotype. As determined by flow cytometry (Fig. 4B–D), the proportion of M2-type macrophages (F4/80 and CD206-positive) cultured with Ti@IL4-HQ and Ti@BMP2/IL4-HQ was 48.7% and 51.6%, respectively, which is significantly higher than that in the control (21%) and Ti group (20.9%). These results demonstrated IL-4 delivered Ti@HQ scaffolds could noticeably increase the polarization of BMDMs toward M2-type. And this effect was further demonstrated by determining the level of pro-/anti-inflammatory cytokines and upstream transcription factors. In comparison with Ti scaffold alone, biomimetic HQ conspicuously down-regulated the expression of mRNA encoding IRF5, which was further decreased by the treatment of Ti@IL4-HQ and Ti@BMP2/IL4-HQ (Fig. 4E). Compared with Ti and Ti@HQ scaffolds, Ti@BMP2-HQ and Ti@BMP2/IL4-HQ down-regulated the expression of pro-inflammatory cytokine IL-1β and IL-6 (Fig. 4F and G). It is well established that IL-4-mediated polarization of M2-type macrophages is through the JAK-STAT6 pathway [38]. The introduction of IL-4 resulted in a significant increase in the gene expression of transcription factor STAT6 and M2-type macrophage-related cytokine IL-10 (Fig. 4H and I). It was previously reported that BMP-2 not only has an osteogenic effect, but also has an immunomodulatory effect, which can up-regulate the expression of IL-10 in M2-type macrophages [39]. Therefore, the treatment of Ti@BMP2-HQ also showed efficacy in M2 macrophage polarization. To further identify the signaling pathway for macrophage polarization and osteogenesis differentiation effects of Ti@BPM2/IL4-HQ, the expression of signal transduction associated proteins was detected by western blot. We found that the expression level of JAK was significantly increased upon Ti@IL4-HQ and Ti@BPM2/IL4-HQ treatment (Fig. 4J), while the expression of p-JAK1 was obvious change. Meanwhile, the activator of transcription 6 (STAT6), a downstream protein of the JAK pathway, was also significantly up-regulated (Fig. 4I), which was in well accordance with previous report that IL-4-induced M2 macrophage polarization is through the JAK-STAT6 pathway [38]. It was also suggested that the use of hydrogel did not affect the bioactivity of cytokines. The protein expression level of RUNX-2, COL I, ALP and OCN genes in Ti@BPM2-HQ and Ti@BPM2/IL4-HQ-treated groups was significantly up-regulated in comparison with other treatments (Fig. 4J). The mechanism of M2-type macrophage polarization and osteogenic gene activation by Ti@BMP2/IL4-HQ are shown in Fig. 4K.

### *In vivo* bone regeneration induced by Ti@HQ composite in femoral defect models

In the bone regeneration process, M2-type macrophages have also been shown to secrete growth factors including BMP-2, BMP-4 and TGF‐β1 that could stimulate the differentiation and activation of pre-osteoblastic cells, boosting the bone mineralization [40]. Of these growth factors, BMPs are essential for bone formation. In the early stage of bone formation [41], BMP-2 can not only recruit undifferentiated mesenchymal stem cells to the bone formation center and facilitate their differentiation into bone lineage cells, but also can reverse the differentiation of fibroblasts, myoblasts and bone marrow basal cells into bone lineage cells [42, 43]. However, the half-time of BMP-2 is short and BMP-2 is also easily degraded *in vivo*. Thus, diverse delivery systems have been used to extend the retention of BMPs to a time period ranging from 1 week to a few months [44].

**Figure 5.**
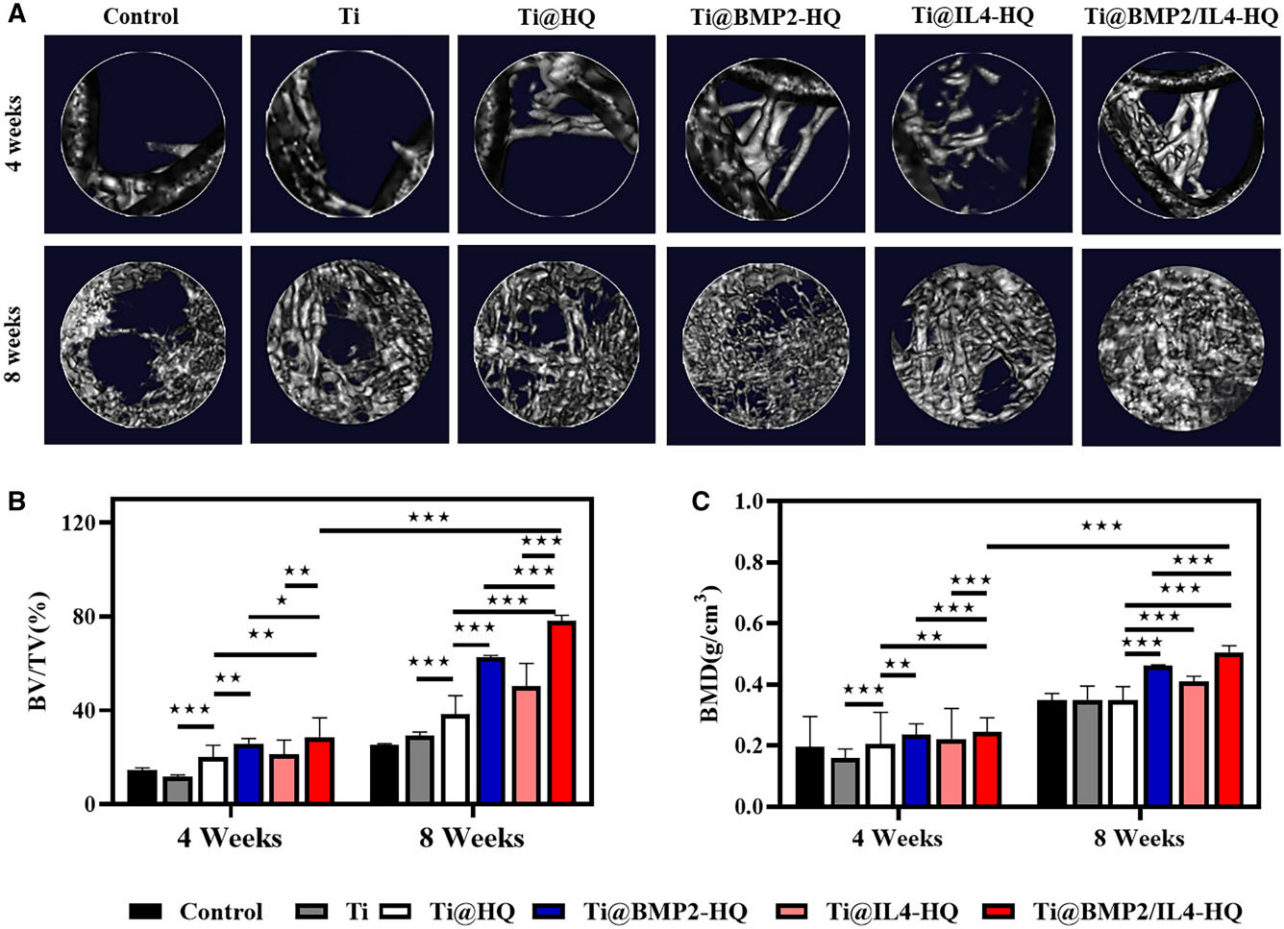
*In vivo* bone regeneration analyzed by Micro-CT imaging. (A) Representative Micro-CT images captured at 4- and 8-weeks post scaffold implantation. The untreated bone defect served as the control group. (B and C) Quantitative analysis of bone volume (B) and bone density (C) within the bone defect area. Data are shown as mean ± SDs (*n* = 8). *P < 0.05, **P < 0.01 and ***P < 0.001, between the indicated groups

Given that Ti@BPM2/IL4-HQ can effectively promote osteogenic differentiation and polarize the macrophage into M2-type, femoral defect models in rat were established and hybrid scaffolds were implanted to induce the bone regeneration. During the experiment period of 8 weeks, no rat died due to complications such as inflammatory soft tissue swelling or heterotopic bone formation. In order to assess the state of the new bone and the degree of osseointegration in the defect area, Micro-CT imaging was performed at 4- and 8-weeks post scaffold implantation. Figure 5A indicated that newly formed bone was clearly observed in the original defect area with various treatments. Compared with Ti alone, more bone tissue was formed around Ti@HQ, suggesting the hybrid of HQ hydrogel can improve the efficiency of bone regeneration by providing a suitable microenvironment for cell infiltration and proliferation. Moreover, *in situ* release of growth factors and cytokines from Ti@BMP2/IL4-HQ further improved the bone regeneration, in comparison with Ti@BMP2-HQ or Ti@IL4-HQ. At scheduled time points (Weeks 4 and 8, Fig. 5B and C), in comparison with the control and other treatment groups, the percentage of bone volume per total volume (BV/TV) and the value of BMD in Ti@BMP2/IL4-HQ group were both significantly higher, which also clearly showed a time-dependent behavior. As the healing time was extended to 8 weeks, the bone volume and BMD were significantly increased. For instance, the value of BV/TV in Ti@BMP2/IL4-HQ-treated defect was increased by 2.63-fold at Week 8 post surgery, in comparison with that at Week 4.

**Figure 6.**
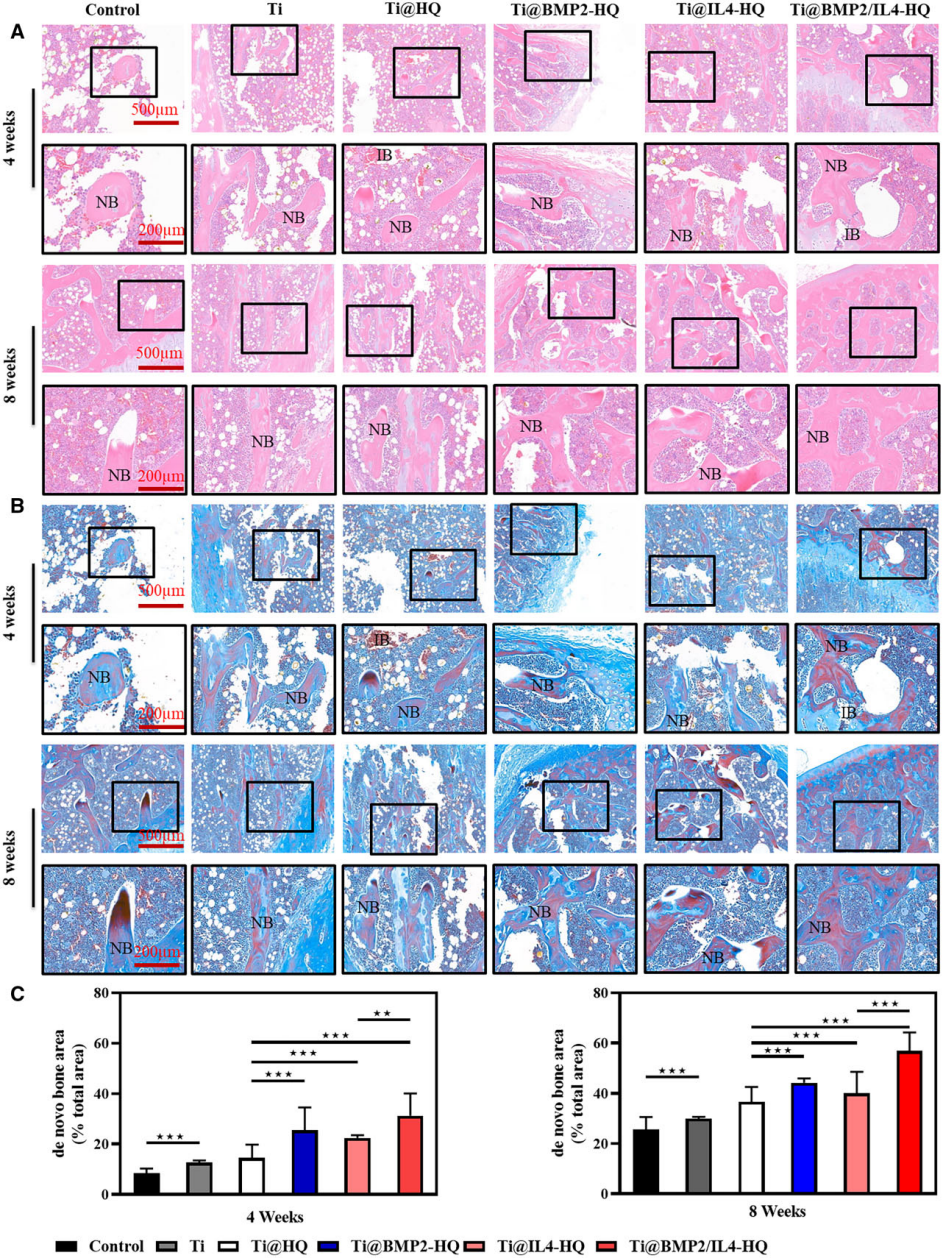
Histological analyses of femur defects at 4- and 8-weeks post treatment. (A and B) Representative images for H&E (A) and Masson’s trichrome (B) staining. IB, immature bone; NB, new bone. (C) Semi quantification of *de novo* bone area. Error bars indicate standard deviation (*n* = 8); **P < 0.01, ***P < 0.001, between indicated groups (Student’s t-test) or compared with all other treatments (one-way ANOVA)

Furthermore, H&E and Masson’s trichrome staining in Fig. 6 demonstrated that new bones were visible in all groups. Ti@HQ treatment resulted in a larger volume of regenerated bone tissues, compared with the control or Ti alone. Moreover, in comparison with BMP-2 or IL-4-encapsulated Ti@HQ scaffold, thicker soft tissues and regenerated bones similar to natural bones were observed with the implantation of Ti@BMP2/IL4-HQ. Besides, the defect area in Ti@BMP2/IL4-HQ-treated bone was correspondingly reduced. At Week 8, immature bones were significantly reduced, and the formation of new bone was significantly improved.

**Figure 7.**
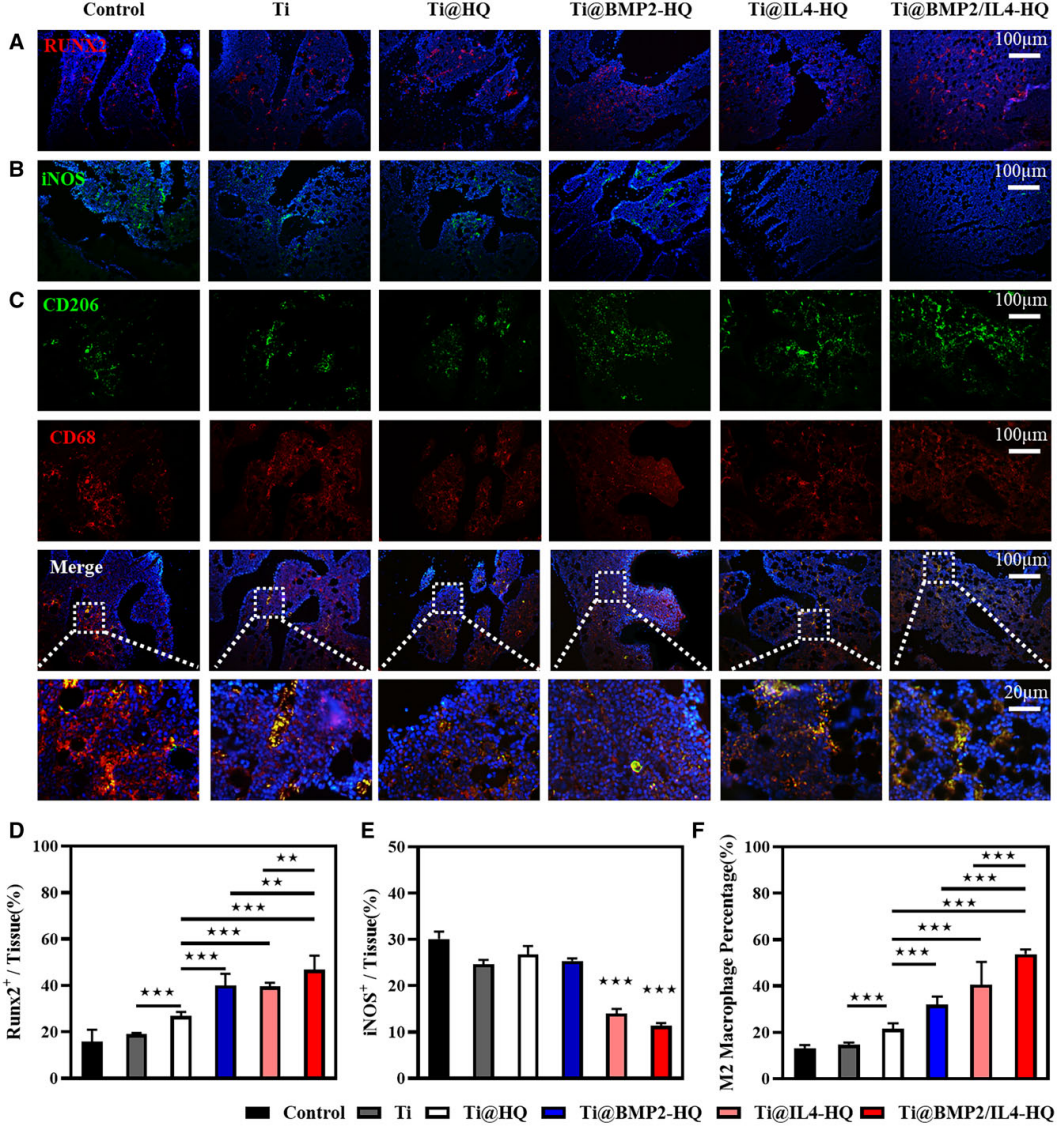
Immunofluorescence staining of osteoblasts and macrophages at the new bone area. (A–C) Representative immunofluorescence images for surface markers including RUNX2 (A, red, osteogenic marker), iNOS (B, green, M1 phenotype) and CD206 (C, green, M2 phenotype) and CD68 (red, macrophages). (D) Semiquantitative results of RUNX2-positive area/tissue area (RUNX2^+^/tissue). (E) semiquantitative results of iNOS positive area/tissue area (iNOS^+^/tissue). (F) The quantitative analysis of CD206- and CD68-positive areas in sectioned new bone tissues at 4 weeks post-operation. Error bars indicate standard deviation (*n* = 3); **P < 0.01, ***P < 0.001, between indicated groups (Student’s *t*-test) or compared with all other treatments (one-way ANOVA)

Immunofluorescence staining was implemented to examine RUNX2-expressing osteogenic cells and the infiltration of inflammatory cells at the new bone area at 4 weeks post treatment. iNOS enzymes and CD206 was used as typical maker for M1 and M2 phenotype macrophages, respectively, while CD68 was used to label macrophages. As shown in Fig. 7A, Ti@BMP2/IL4-HQ group showed a more widespread distribution of RUNX2-positive cells (red fluorescence), in contrast to other groups. Semiquantitative determination of RUNX2-positive area to tissue area in vision field (RUNX2^+^/tissue) shows that this ratio for Ti@BMP2/IL4-HQ was ∼47%, which was significantly higher than that in other groups (Fig. 7D). The percentage of M1 macrophages (iNOS^+^) at the regenerated tissue treated by Ti@BMP2/IL4-HQ (11.3%) was lower than that with the treatment of Ti@IL4-HQ (14%), Ti@BMP2-HQ (25.3%) and Ti@-HQ (26.7%) (Fig. 7B and E). Meanwhile, as shown in Fig. 7C and F, the ratio of M2-type macrophages indicated by CD206/CD68 staining for Ti@BMP2/IL4-HQ group was 53.7%, which was significantly higher than that in Ti@BMP2-HQ (32%, *P* < 0.001) and Ti@IL4-HQ (40.6%, *P* < 0.001) groups. These results indicated that Ti@BMP2/IL4-HQ potently stimulated the macrophage polarization to M2 type in femur defects, providing an osteoimmunomodulatory effect on bone formation, which was also further evidenced by immunochemistry staining (Fig. S14) of the regenerated bone tissues. These data suggested that *in situ* release of BMP-2 and IL-4 at the scaffold–bone interface using Ti@HQ composite scaffold noticeably induced the osteogenesis at the defective location and promoted the bone regeneration.

### Antibacterial function characterization *in vitro* and *in vivo*

**Figure 8.**
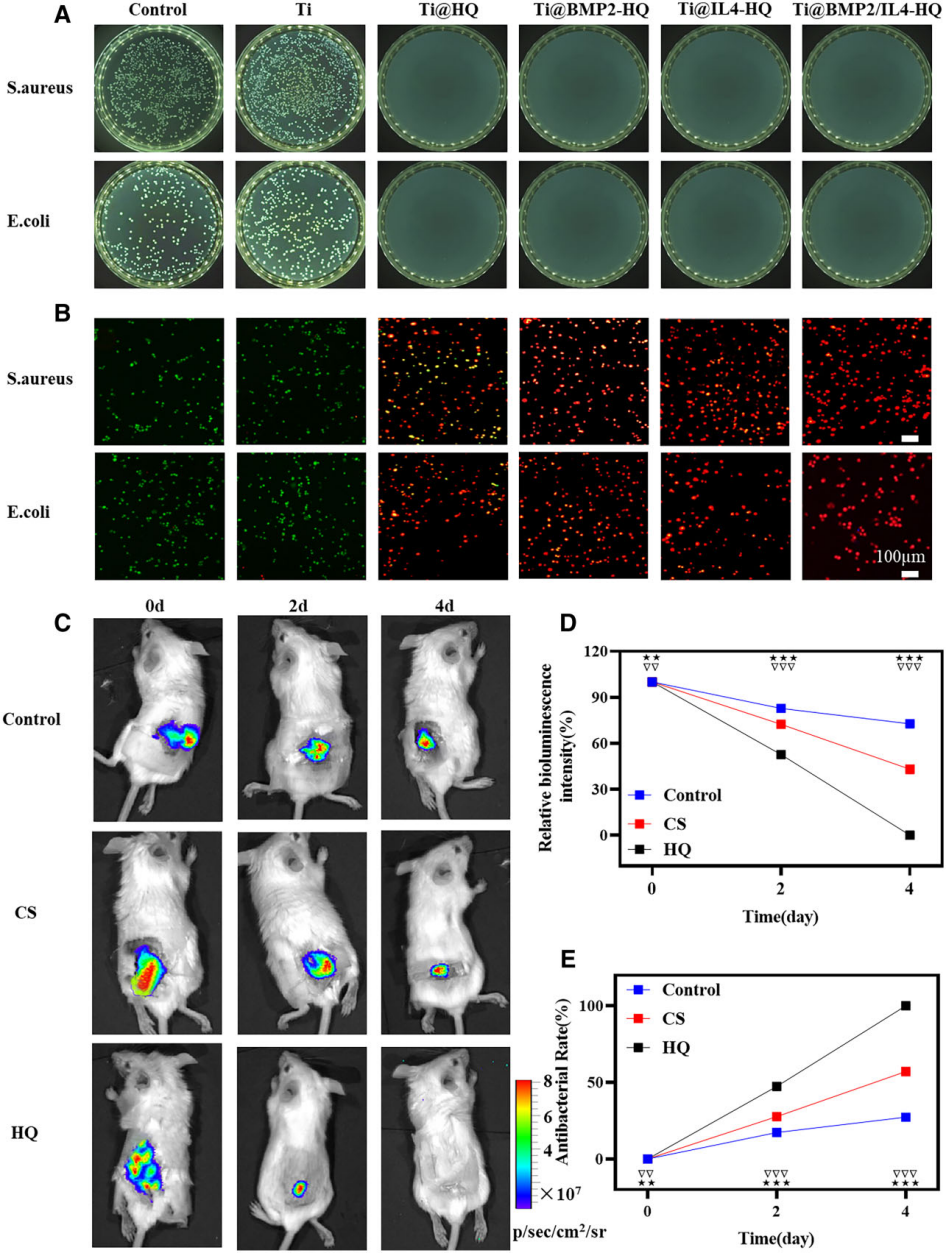
Antibacterial function of Ti@BMP2/IL4-HQ scaffold *in vitro* and HQ *in vivo*. (A) Representative photographs for bacteria colonies in agar plates. (B) Fluorescence microscope images of *S. aureus* and *E. coli* cultured on the scaffold for 12 h and stained by Live/Dead assay. Green channel, live bacteria; red channel, dead bacteria. (C) Time-dependent bioluminescence images of infected mice treated with saline, commercial CS and HQ hydrogel. (D) The relationship between the relative bioluminescence intensity and time. The bioluminescence intensity before treatment (Day 0) was set as 100%. (E) Antibacterial ability calculated by the attenuation of the bioluminescence signals. Error bars indicate standard deviation (*n* = 3); **P < 0.01 and ***P < 0.001, HQ versus control or CS; ^##^*P* < 0.01 and ^###^*P* < 0.001, antibacterial capacity at present time point versus all that at previous time points for the HQ group

Bacterial-induced infection remains a major challenge in bone healing [45]. An effective approach to address this issue is endowing the scaffold with inherent antibacterial capability. Thus, CS was conjugated with quaternary ammonium salt to confer the hydrogel inherent antimicrobial ability. Gram-positive *S. aureus* and Gram-negative *E. coli* were used as representative bacteria strains to evaluate the antibacterial effect of HQ. Results in Fig. 8 showed that no obvious colony was found for *S. aureus* and *E. coli* treated by Ti@HQ and other composite scaffolds for 12 h (Fig. 8A), while there were a large number of bacteria colonies in Ti implant and the control group. In addition, fluorescence microscopy for live/dead staining further confirmed the sterilization ability of HQ (Fig. 8B). A large number of alive bacteria (green) were observed in the control and Ti groups; however, the treatment of HQ resulted in the extensive death of bacteria (stained as red). The bactericidal activity of HQ was mainly attributed to the positively charged quaternary ammonium salt in QCS, which could rupture the bacterial membrane, inducing the leakage of biologically active molecules and the death of bacteria [46]. Then, the antibacterial activity of HQ *in vivo* was further assessed against bioluminescent *S. aureus Xen 36*. Compared with the treatment of saline or commercial CS hydrogel, HQ significantly reduced the bioluminescence intensity of *S. aureus Xen 36* with a time-dependent profile (Fig. 8C). The attenuation of bioluminescence signals (Fig. 8D and E) in the infected area also proved that HQ possessed strong bacterial killing effect. Four days later, initial bacteria inoculated at the infection site was completely cleared with the treatment of HQ hydrogel, which was conspicuously superior than CS hydrogel. These results indicated that HQ was effective in eliminating bacteria in the infected area, holding great potential to protect the scaffold from bacterial invasion and prevent acquired infection during the formation of new bone.

## Discussion

Hybrids of organic polymers with or without growth factors with metal scaffold have also been leveraged to repair bone injures [47–51]; however, these coatings were mainly designed to regulate the surface physiochemical characters of metal implants or to deliver drugs for enhancing either vascularization or osteogenesis [39, 52, 53]. The osteoinduction of implants and regulation of immune microenvironment at the injured tissue remain to be further improved for bone regeneration. Hydrogel is widely used for tissue regeneration because its molecular structure is similar to the natural ECM in the organ or tissue, providing spatial structure for cell infiltration and proliferation. In addition, hydrogel could also deliver nutrients to the living cells surrounding the defect tissue. Here, polysaccharide hydrogel was composited with 3D-printed titanium alloy with superior mechanical support that is required for bone repair. Compared with polymer hydrogels previously reported for bone regeneration, the hybrid polysaccharide hydrogel could mimic the chemical components and porous network of tissue ECM, serving as a matrix for cell adhesion and tissue growth. It was found that Ti@HQ scaffold without cytokines or growth factors could also facilitate the bone regeneration, suggesting that Ti@HQ could be leveraged to explore a cytokine-free approach for the reconstruction of bone defects by optimizing the composition, mechanical properties and biological function of HQ hydrogel.

Recently, macrophages have been identified as an effective target for immunomodulation in tissue regeneration [54–56]. Biomaterials such as hydrogels that can mimic ECM and thereby provide a favorable microenvironment for macrophage polarization, could expedite the regeneration of defected tissues on their own [57–59]; however, the efficacy of tissue regeneration was limited, in comparison with cytokine therapy. The hydrogel reported here also acted as a cytokine depot for M2-type macrophages polarization and osteoinduction to accelerate bone regeneration. Results shown in the present study demonstrated that the priming of host macrophages to M2 phenotype though sustained delivery of IL-4 was able to promote the osteoblast differentiation and proliferation of hBMSCs. Such a synergy resulted in effective repair of large-scale femoral defect by the hydrogel–metal implant composite. To our best knowledge, such a bioactive composite scaffold was not reported previously. Moreover, materials and cytokines used in this work are FDA-approved or commercially available, conferring the composite scaffold great promise for clinical translation.

Concerning the cytokine delivery, diverse delivery systems have been used to extend the retention of BMPs at the bone defect site, which is necessary to allow hBMSCs to migrate to the injury area. Major delivery systems for BMPs include natural polymers, inorganic materials, synthetic polymers, and the composites of these materials, which could result in a wide range of retention profiles of BMPs varying from 1 week to a few months [44]. Commonly, the addition of a delivery system significantly increased the recombinant human bone morphogenetic protein-2 (rhBMP-2) retention compared to that in buffer solution alone and the release time period of BMP depends on the degradation rate of the carrier [60]. Specially, the release time period of rhBMP-2 from hyaluronan-based delivery system is 1–2 weeks [61]. Meanwhile, delivery system with low degradation rate, such as the calcium phosphate cement, can prolong the rhBMP-2 retention profile to 4–5 weeks [62]. This study indicated that HQ hydrogel incorporating low-dose BMP-2 promoted the recruitment of osteoprogenitor cells to the defect site and resulted in robust repair of large-scale femoral defect in rat within 8 weeks. In addition, the immunomodulation by IL-4-induced M2 macrophage polarization also helped the bone regeneration. As a proof-of-concept study, the composite scaffold shows great promise in treating bone defectives including but not limited to femur injury. However, in order to further improve the efficacy of bone regeneration and achieve clinical transformation, it is necessary to optimize the concentration of polysaccharide in hydrogel, the loading amount and controlled release of cytokines, as well as the fabrication procedure of composite scaffolds in future works.

## Conclusions

In summary, a new type of hybrid and chemically crosslinked polysaccharide hydrogel composed of quaternary ammonium CS and HA was successfully developed to non-covalently fill the pores of the titanium alloy scaffold to construct a hydrogel–metal composite scaffold for bone regeneration. The present hybrid hydrogel holds inherent merits including ECM-mimicking, self-healing property, along with facile manufacture and good biodegradability. The dynamic imine bond confers the HQ hybrid hydrogel self-repairing, injectable and biodegradable properties necessary for tissue repair. Moreover, endogenous growth factors and cytokines could be persistently released from the hydrogel surrounding the Ti scaffolds, which could modulate the pro-regenerative immune microenvironment for accelerating the bone regeneration by simultaneously boosting the polarization of macrophages to M2-type and the differentiation of hBMSCs to osteoblasts. In addition, the hydrogel showed high antibacterial efficacy against both Gram-positive and Gram-negative bacteria strains, providing potential in eliminating infection to the scaffold or acquired infection during bone regeneration. Overall, the design and manufacturing of bioinspired hydrogel–metal composite presents a promising strategy for preparing bioactive scaffold that can tremendously improve the treatment of bone injures in clinic.

## Supplementary data

Supplementary data are available at *REGBIO* online.

*Conflict of interest statement*. None declared.

## Funding

This work was financially supported by the National Natural Science Foundation of China (32171380, 31971306), Natural Science Fund for Distinguished Young Scholars of Tianjin (21JCJQJC00020), CAMS Innovation Fund for Medical Sciences (No. 2021-I2M-1-065, 2021-I2M-1-058), Non-profit Central Research Institute Fund of Chinese Academy of Medical Sciences (NO. 2019-F40-SYS) and Tianjin Innovation and Promotion Plan Key Innovation Team of Implantable and Interventional Biomedical Materials.

## Supplementary Material

rbac003_Supplementary_Data
