# Supplementary material for: Titanium alloy composited with dual-cytokine releasing polysaccharide hydrogel to enhance osseointegration via osteogenic and macrophage polarization signaling pathways
Source: Regen Biomater. 2022 Jan 12;9:rbac003. doi: 10.1093/rb/rbac003 (PMC9160882; doi:10.1093/rb/rbac003)
Supplement: rbac003_Supplementary_Data [file rbac003_supplementary_data.doc]

Supplementary data for “Titanium alloy composited with dual-cytokine releasing polysaccharide hydrogel to enhance osseointegration via osteogenic and macrophage polarization signaling pathways”

Yaping Wang ^a, b, 1^, Zujian Feng ^b, c 1^, Xiang Liu ^a, b^, Chunfang Yang ^b, c^, Rui Gao ^b, c^, Wenshuai Liu ^b^, Wenbin Ou-Yang ^b, d^, *, Anjie Dong ^a^, *, Chuangnian Zhang ^b, c^, Pingsheng Huang ^b, c^, Weiwei Wang ^b, c^ *

^a^ Department of Polymer Science and Engineering Key Laboratory of Systems Bioengineering (Ministry of Education) School of Chemical Engineering and Technology Tianjin University, Tianjin 300072, China

^b^ Key Laboratory of Innovative Cardiovascular Devices, Chinese Academy of Medical Sciences

^c^ Tianjin Key Laboratory of Biomaterial Research Institute of Biomedical Engineering Chinese Academy of Medical Sciences and Peking Union Medical College, Tianjin 300192, China

^d^ Structural Heart Disease Center, National Center for Cardiovascular Disease, China and Fuwai Hospital, Chinese Academy of Medical Sciences and Peking Union Medical College, Beijing 100037, China

^1^ These authors contributed equally to this work.

* Corresponding authors:

Prof. Dr. Anjie Dong, Email: ajdong@tju.edu.cn

Prof. Dr. Wenbin Ou-Yang, Email: droywb31@163.com

Prof. Dr. Weiwei Wang, Email: wwwangtj@163.com, wangww@bme.pumc.edu.cn

**SUPPLEMENTARY MATERIALS AND METHODS**

**Materials**

Glycidyl trimethylammonium chloride (GTMAC), sodium periodate, ethylene glycol, hydroxylamine hydrochloride, sodium periodate (NaIO4) and acetylene glycol hydroxylamine hydrochloride were purchased from Aladdin Chemical Reagent Co., Ltd. (Shanghai, China). BMP-2, IL-4 and recombinant mouse monocyte colony stimulating factor (M-CSF) were purchased from Pepro Tech (Rocky Hill, NJ, USA). Fluorescent dye-labeled monoclonal antibodies against CD68, CD206 and F4/80 were provided by eBioscience (San Diego, California, USA). LPS, DAPI and Actin-Tracker Red-555 were purchased from Beyotime Biotechnology (Shanghai, China). The primary and secondary antibodies for CD68, CD206, RUNX2, iNOS, IL-4, IL-1β, IL-6, ALP, COL I, OCN, Runx2, JAK1, p-JAK1, and β-actin were purchased form Abcam (Cambridge, UK). Alizarin Red, Sirius Red staining, Rhodamine B and Elisa kits were purchased from Sigma-Aldrich. SYTO-9 green fluorescent nucleic acid stain and propidium iodide (PI) were purchased from Gibco (USA), and all other chemical agents and solvents were analytical grade. hBMSCs are provided by Research Center for Human Tissue and Organs Degeneration, Institute Biomedical and Biotechnology at Shenzhen Institute of Advanced Technology, Chinese Academy of Sciences. The commercially available E. coli (ATCC 25922), S. aureus (ATCC 6538) were purchased from American Type Culture Collection, Manasses, USA. The commercially available bioluminescent S. aureus Xen36 was purchased from PerkinElmer Inc. (Waltham, MA, USA).

**Experimental Section**

*Characterization of QCS and HA-CHO:* The degree of oxidation was determined by colorimetric analysis and ^1^H-NMR (Varian 400 spectrometer, Varian, USA). The colorimetric hydroxylamine titration was performed by a potentiometric titrator (ZEDJ-4B automatic potentiometric titrator, INESA Scientific Instrument Co., Ltd., China). In short, HA-CHO (100 mg) was dissolved in hydroxylamine hydrochloride (25 mL, 0.25N, pH adjusted to 3) to consume aldehydes and hemiacetals. The mixture was titrated with NaOH (0.1 N) after stirring continuously for 4 h. The percentage of aldehyde functionality was calculated by: aldehyde functionality (%) = mol of dialdehyde/ mol of HA × 100 = V_NaOH_ × C_NaOH_ / (m_HA_ / (Mn_HA_-18)) × 100, where V_NaOH_, C_NaOH_, m_HA_ and Mn_HA_ represent the volume of NaOH solution, the concentration of NaOH solution, the mass of HA and the number-average molecular weight of HA, respectively. The molecular weight of HA-CHO was measured by GPC. The system is equipped with PLgel GPC column (10 µm Mixed-B, Org 300 × 7.8 mm) and RI2000 detector.

FT-IR spectroscopy (Elmer Perkin, USA) was used to characterize HQ polymer. In order to determine the formation of HQ, the sol-gel transition was determined by tube inverting method. AR 2000ex rheometer (TA) with a circulating environment system for temperature control was used to study rheological properties of hydrogels. HQ was placed between parallel plates with a diameter of 25 mm and a gap of 0.5 mm. The elastic modulus (G') and viscosity modulus (G'') were measured in a frequency range of 0.1 to 100 rad/s and a time range of 15 minutes.

The internal structure of HQ was studied by scanning electron microscopy (SEM) (S-4800, Hitachi, Japan). HQ was frozen in liquid nitrogen and then lyophilized. At least three random areas were captured for each sample to analyze the morphology of HQ.

Self-healing features of HQ were examined. The HQ and RB-labeled HQ were cut into two pieces respectively, and then half of the HQ and half of the RB-labeled HQ were incubated together to observe their reformation. The reformed HQ was incubated in 0.01 M PBS (pH = 7.4) for 24 h to evaluate its stability. The photos of each step were captured by a digital camera. The self-healing property of HQ was also tested by investigating the changes of G' and G'' under continuous strain sweep with an alternative large oscillation force (50%) and a small one (2%). To determine the adhesion of HQ, the bonding strength between HQ and substrates including glass, wood, plastic, silicone and titanium plates was measured by material testing instrument (Instron 3360, America).

To assess the swelling of HQ, samples were immersed in 50 mL PBS for 24 h. The swelling ratio was quantified by equation: swelling ratio = (W_s_ - W_d_)/W_d_, where W_s_ and W_d_ refer to the swollen and dry weights, respectively.

For degradation study *in vitro* HQ (0.5 g) was incubated in PBS (0.01 M, pH = 7.4) at 37 °C and the medium was totally replaced once a day by fresh PBS. HQ was cultured in medium in the presence of hyaluronidase (300 μg/mL) and the weight of remaining hydrogel was monitored. The decay of HQ mass was calculated within a time period of two weeks.

The release of BMP-2 and IL-4 from the hydrogel was determined in vitro. In short, BMP2/IL4-HQ (100 ng, 100 ng, 200 µL) was immersed in PBS (200 µL) supplemented with 0.5 U/mL hyaluronidase to simulate the release of factors in the body. The BMP2/IL4-HQ was then incubated at 37 °C. The supernatant was collected and replenished with fresh solution at scheduled time point (0, 2^nd^, 4^th^, 6^th^, 8^th^, 10^th^, 12^th^, 14^th^ day). The collected solution was stored at - 80 °C for analysis. The amount of BMP-2 or IL-4 released in the supernatant was quantitatively determined by enzyme-linked immunosorbent assay (ELISA) according to the manufacturer’s instructions, and the accumulative release was calculated.

*Rhodamine B fluorescent labeling and in vivo imaging after subcutaneous implantation:* The degradation of HQ in vivo was investigated by fluorescence imaging to visualize the gel in a real-time manner. The HA was labeled with rhodamine B (RB) by chemical conjugation according to the previously reported method[1]. RB-HA was then mixed with QCS to form hydrogel according to the aforementioned procedure. Subsequently, HQ (200 µL) was injected subcutaneously at the back of female BALB/c mice (n = 3). The fluorescence signal was detected by the CRI Maestro imaging system (CRI Corporation, Woburn, MA, USA) and quantified using region of interest (ROI) analysis. The total fluorescence signal (TFS) was expressed as mean ± SD (n = 3) at different time points.

*Hemolysis assay:* For hemolysis study, the anticoagulated rabbit blood (2 mL) was centrifuged at 1500 rpm for 5 min to collect the erythrocytes and further rinsed with sterile PBS for three times. Then, QCS, HA-CHO, HQ, BMP2/IL4-HQ (1 mg/mL) were added to erythrocyte suspension. Deionized water and physiological saline were used as positive and negative control groups. After incubated for 1 h at 37 ^o^C, the mixture suspension was centrifuged and recorded the absorbance of the supernatant at 540 nm using a multifunction microplate reader (Varioskan Flash, Thermo Scientific). and the hemolysis ratio was calculated using the following formula:

Hemolysis ratio = (OD_sample_ – OD_negative_)/ (OD_positive_ – OD_negative_) × 100%

*Alkaline phosphatase (ALP) activity:* After culturing for 3, 7 or 14 days, cells were lysed by M-PER reagent (Thermo Scientific, USA). After centrifugation at 2000 rpm for 4 minutes to remove the cell debris, the supernatant (50 μL) was transferred to 96-well plates and mixed with 50 μL of p-nitrophenyl phosphate (p-NPP). The ALP detection reagent was then added. Finally, the absorbance at 405 nm was detected. The total protein in the cell lysate was determined using a bicinchoninic acid (BCA) protein assay kit (Pierce, Rockford, USA). The ALP levels were normalized to the total protein of the samples and the data were expressed as the specific ALP activity per unit of protein. Each sample was analyzed in triplicate.

*ECM mineralization and collagen secretion of hBMSCs*: The ECM mineralization and collagen secretion of hBMSCs were evaluated by Alizarin Red and Sirius Red staining, respectively. After cell culturing for 21 days, Alizarin Red S or 0.1% Sirius Red dissolved in saturated picric acid buffer solution (1 mg/mL) was added and incubated for 30 minutes to visualize the ECM mineralization and collagen secretion, respectively. After carefully washed with deionized water, the stained cells were dried and photographed. The absorbance at a wavelength of 540 nm or 620 nm was measured by a microplate reader.

*Reverse transcription polymerase chain reaction:* To study the effect of the biomimetic scaffold on the differentiation of hBMSCs, the expression of specific genes involved in osteogenesis were quantitatively determined. hBMSCs were seeded on 24-well plates at a density of 1 × 10^5^ cells per well and inoculated with scaffolds for 7 or 14 days. Total RNA was isolated by washing the sample three times with PBS and disrupting with 1 mL TRIZOL. PikoReal 96 Real-time Thermal Cycler (Thermo Fisher Scientific, Finland) was used for quantitative RT-PCR (qRT-PCR) analysis using Real Master Mix (SYBR Green) (NEWBIO, China) and a thermal cycling was performed at 95 °C for 2 min, followed by 45 cycles at 95 °C for 20 s and 58 °C for 20 s, 72 °C for 30 s. The qRT-PCR reactions were performed in triplicates for each sample. Aggrecan was used as the reference gene and the primer sequences were listed in the Supplementary Table 1 (COL I, COL II and CoLX).

*Western Blot:* RIPA lysis buffer (Beyotime, P0013B) was applied to obtain total cell lysates. Primary antibodies against Runx-2 (Abcam, ab92336), COL I (Abcam, ab34710), ALP (Abcam, ab224335), OCN (Abcam, ab133612), JAK1 (Abcam, ab133666), phospho-JAK1(Abcam, ab 138005) were used for protein staining according to the manufacturer’s recommendations. Proteins were extracted, dissolved by SDS-PAGE and then transferred to 0.22 μm polyvinylidene difluoride (PVDF) membranes. The PVDF membrane was blocked with 5 wt% BSA in TBST for 2 h at room temperature and probed with the indicated primary antibodies at 4 ℃ overnight, which were rinsed and incubated with dilutions of appropriate secondary antibodies conjugated with horseradish peroxidase (Cell Signaling Technology) for 1 h at room temperature followed by incubation with an enhanced chemiluminescence kit (Bio-Rad, USA) for a few seconds. Proteins on the membranes were visualized by Chemiluminescent HPR Substrate and images were captured by Chemiluminescence Imaging system (ChemiScope 6000 Pro, China). The signal intensity of immunoreactive bands was quantified by ImageJ software and normalized using β-actin.

*Immunohistochemistry staining*: Immunohistochemistry was carried out using a two-step detection kit. Briefly, specimens were immersed in antigen retrieval solution for 20 min, blocked for 30 min with 5% bovine serum albumin (BSA), and subsequently incubated with primary antibodies against rabbit RUNX2 for osteogenesis markers, for a mononuclear phagocyte recruitment marker, CD68 for a pan-macrophage marker, CD206 for an M2 marker, and iNOS for an M1 marker overnight at 4 °C. After rinsing thoroughly in PBS, the secondary antibodies were dropped onto slides. Images were captured by CLSM and quantitatively analyzed by Image J software.

*Antibacterial test:* Bacterial solution (0.2 mL, 1 × 10^6^ CFU/mL) was mixed with the Ti, Ti@HQ, Ti@BMP2-HQ, Ti@IL4-HQ and Ti@BMP2/IL4-HQ. After incubating at 37 °C for 12 h, PBS (10 mL) was added and the mixture was processed by vortex for 15 seconds. Then the bacterial solution was collected and the bacterial suspension (20 μL) was evenly spread on the agar plate and the active bacteria were counted. The antibacterial rate (AR) was determined by the following formula:

where N_1_ and N_2_ were the counts of bacteria from the control and experimental groups, respectively.

*Antibacterial study in vitro:* The bactericidal efficiency of the composite scaffold was further checked by live/dead fluorescent staining with SYTO-9 (green fluorescent nucleic acid dye) and propidium iodide (PI, red fluorescent DNA dye). After co-cultivation with the bacteria suspension with a concentration of 1 × 10^6^ CFU/mL for 12 h, the scaffold was gently washed 3 times with PBS. Subsequently, live/dead staining was performed and bacteria were imaged by a fluorescence microscope.

*Antibacterial study in vivo:* BALB/c mice were randomly divided into 3 groups (5 mice in each group), and each mouse was received subcutaneous injection of 100 µL suspension of bioluminescent *S. aureus Xen 36* (10^8^ CFU/mL). Then, 200 µL of commercial chitosan (CS) or HQ hydrogel was injected. *In vivo* bioluminescence imaging was employed to record the bioluminescence intensity of bacteria.


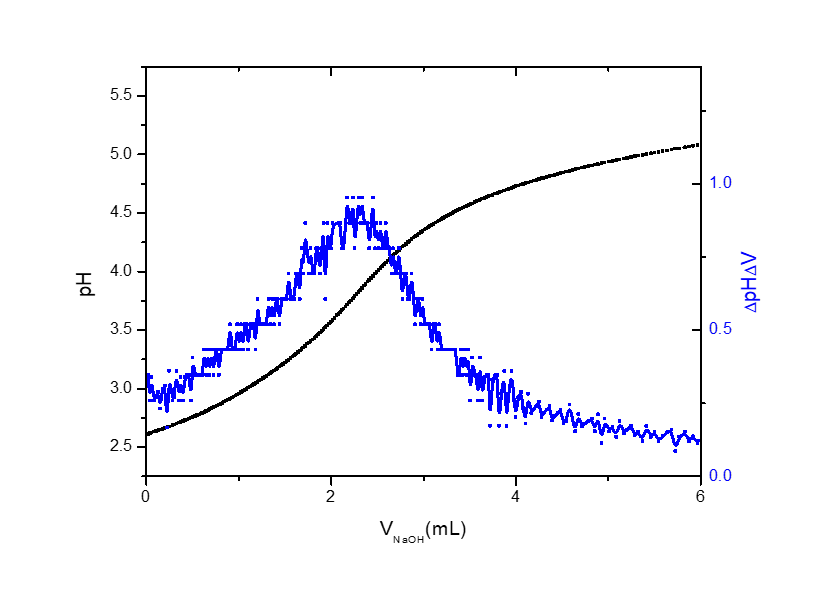


**Figure S1**. Colorimetric hydroxylamine titration analysis of HA oxidation. Black line: HA-CHO titration with hydroxyl-amine. Blue dash line: the first derivative of the titration was utilized to determine the equivalence point.


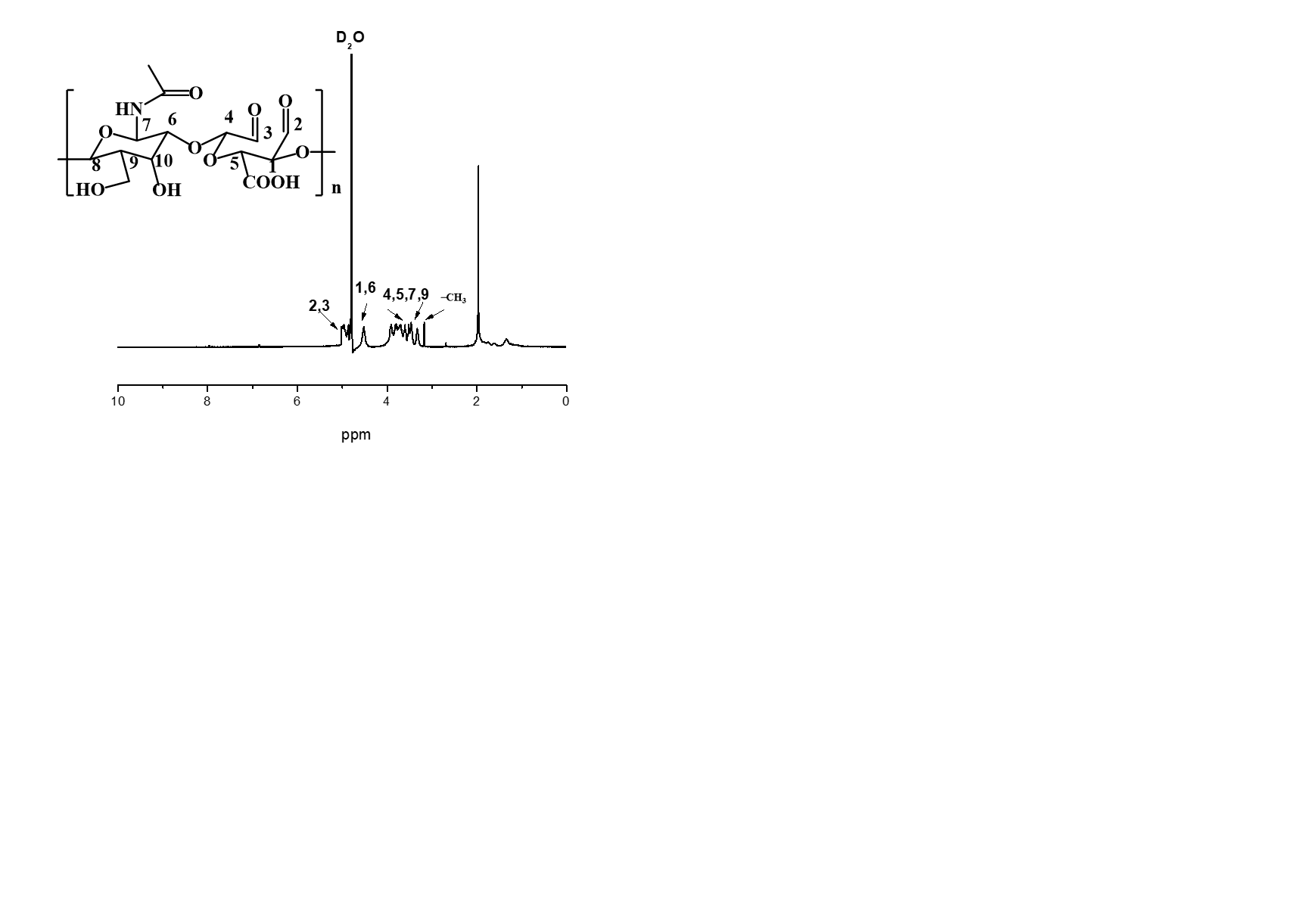


**Figure S2**. The ^1^H NMR spectra of oxidized HA (HAO).


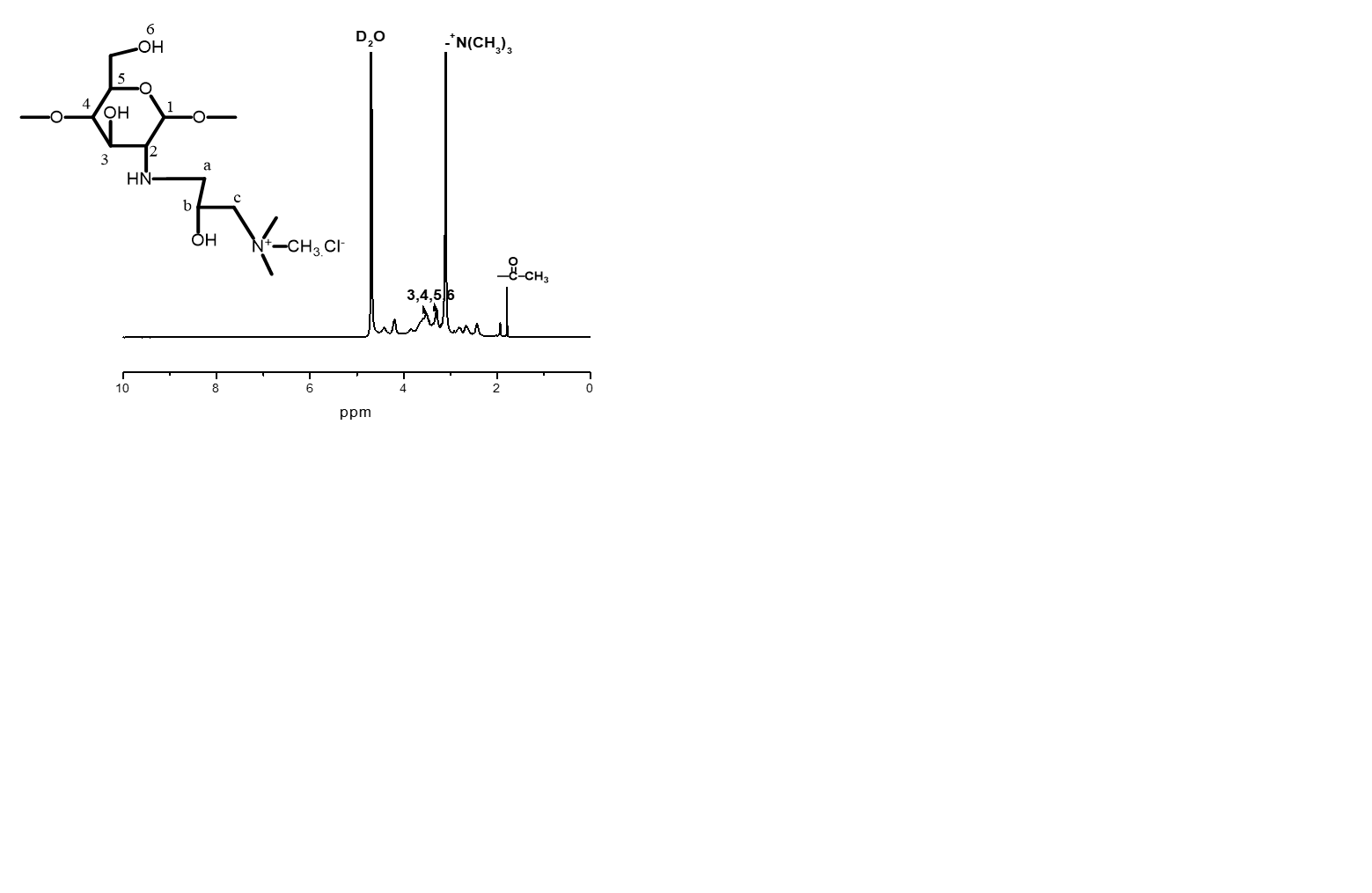


**Figure S3**. The ^1^H NMR spectra of quaternized chitosan (QCS).


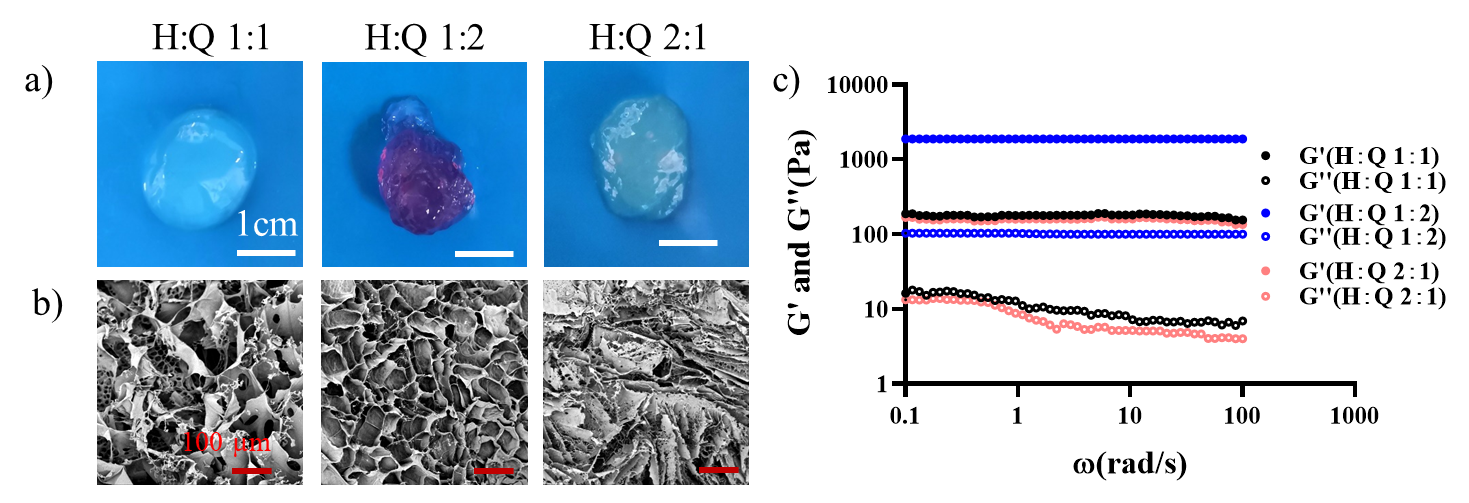


**Figure S4**. (a, b) Photographic (a) and SEM (b) images for HQ hydrogels at various volume ratios. (c) Strain-dependent modulus change for HQ hydrogels.

**Figure S5**. The FI-TR spectrum of CS (a), QCS (b), HA (c), HA-CHO (d) and HQ (e).


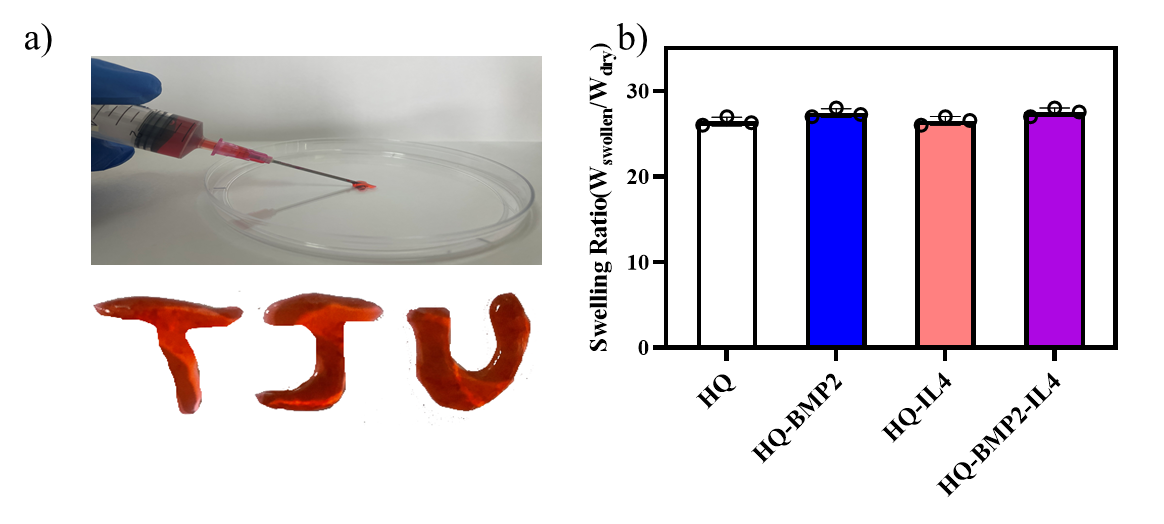


**Figure S6**. (a) Photographic images of HQ demonstrating the injectability. (b) The swelling rate of hydrogels.


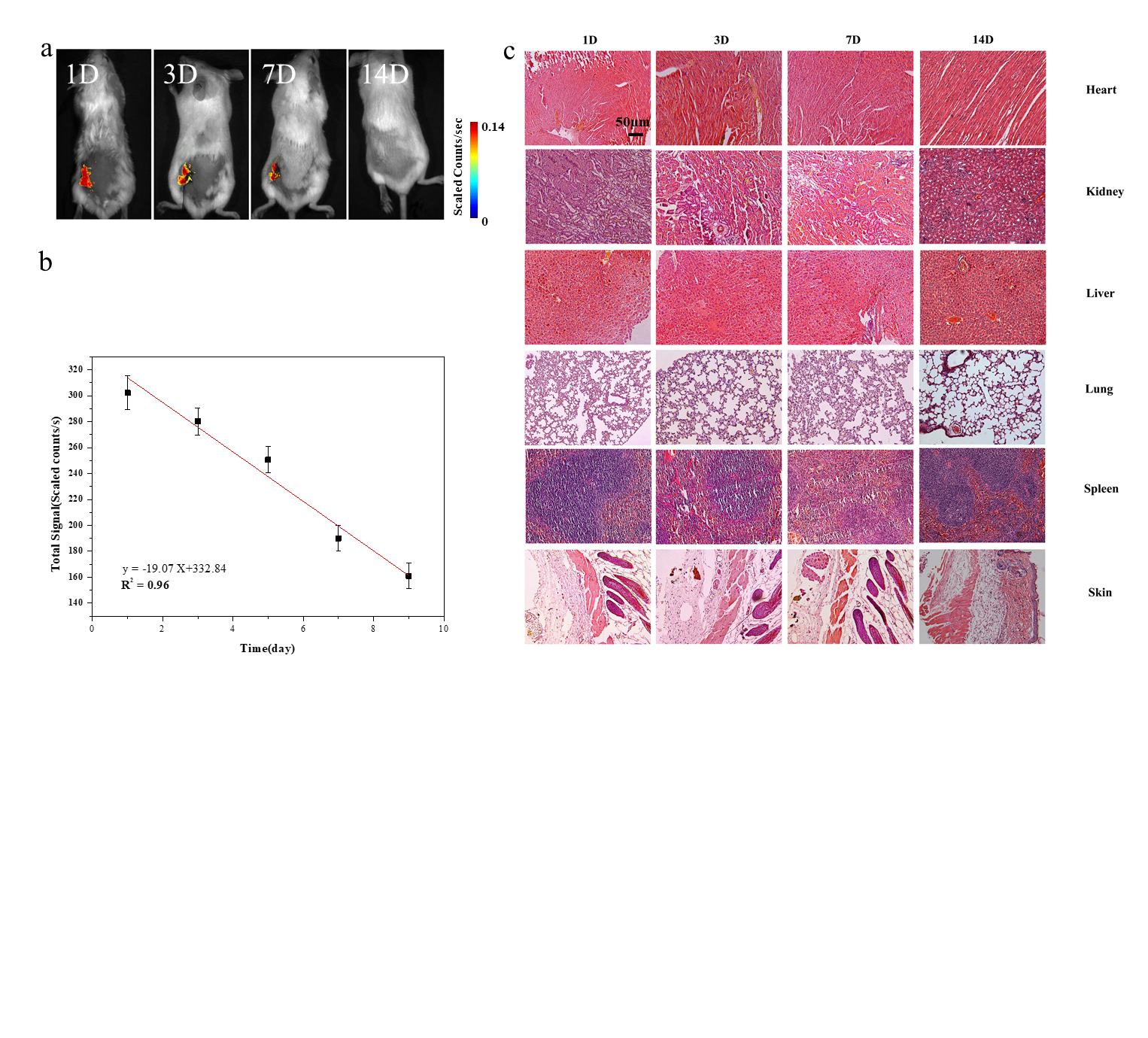


**Figure S7**. (a) Time dependent fluorescent images of mice received subcutaneous injection with RB-labeled HQ hydrogel. (b) The fluorescence intensity of RB at different time points after injection. (c) H&E staining images of liver, heart, spleen, lung, kidney and skin.


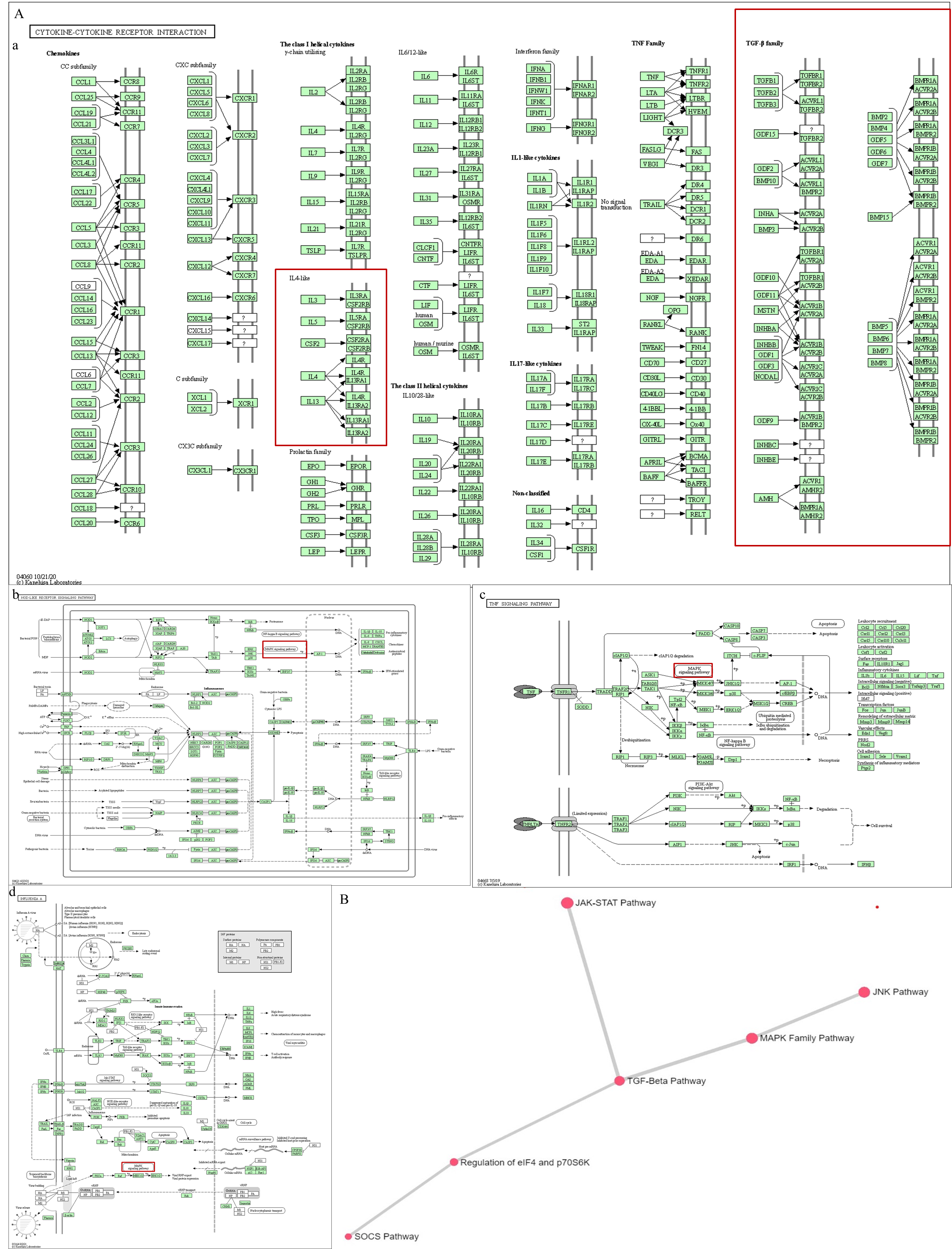


**Figure S8**. (A) Cytokine–Cytokine receptor interaction pathway, Influenza A pathway, NOD-like receptor interaction pathway, and TNF signaling pathway downloaded from DAVID database. Genes marked in red were IL-4-like and TGF-β family (a). MAPK signaling pathway was also marked in red (b-d). (B) The interaction among JAK-STAT, TGF-Beta, and MAPK family pathways.


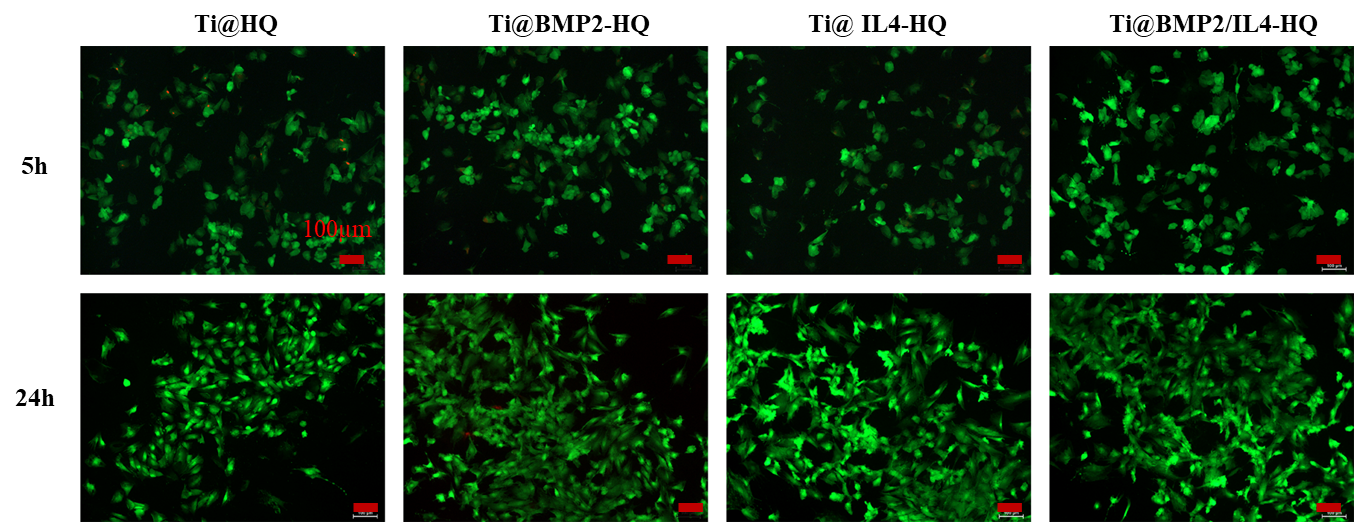


**Figure S9**. Live/dead staining of cells incubation for 5 h and 24 h in vitro.

**Figure S10**. The average diameter of cells incubation for 5 h in vitro.


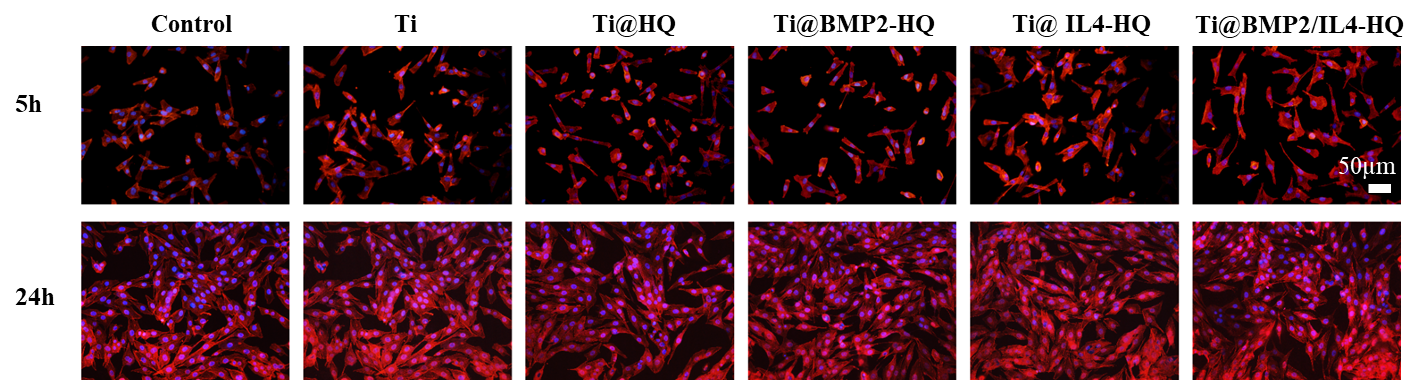


**Figure S11**. Fluorescence images of hBMSCs cultured on various scaffolds.


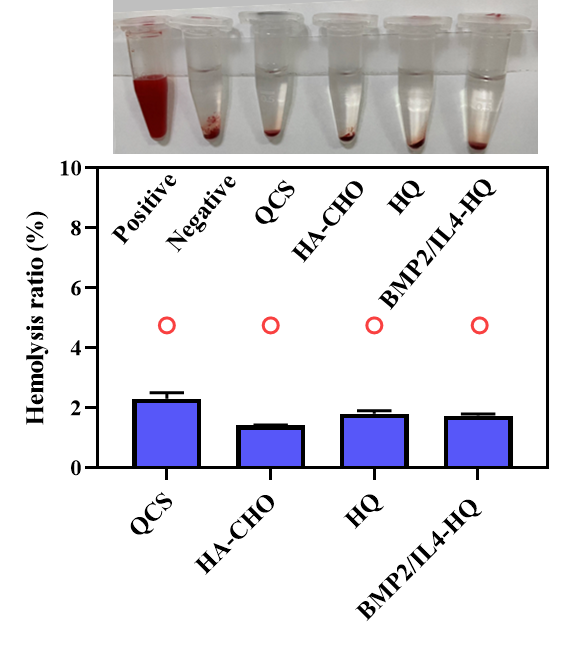


**Figure S12**. Hemolytic properties of QCS, HA-CHO, HQ hydrogel, and BMP2/IL4-HQ hydrogel. The circle represents the hemolysis of 5%.


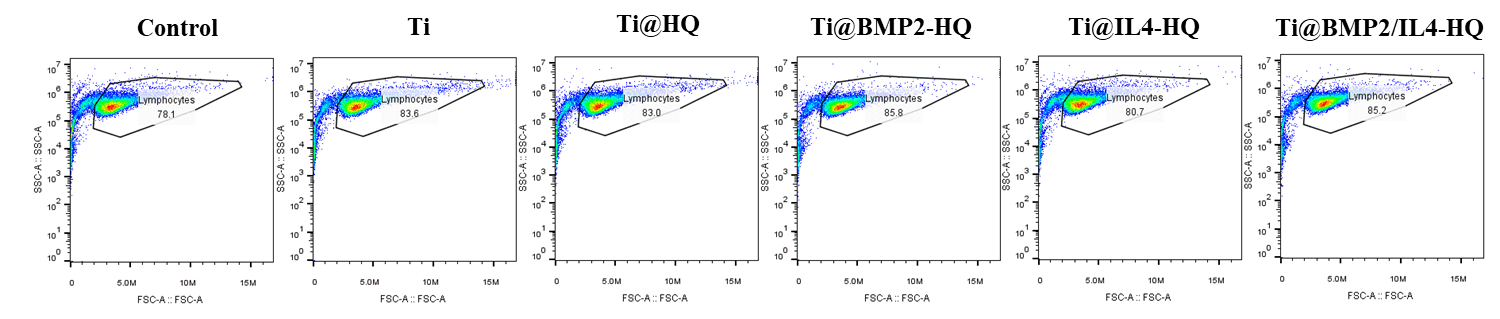


**Figure S13**. The gating principle of flow cytometry.


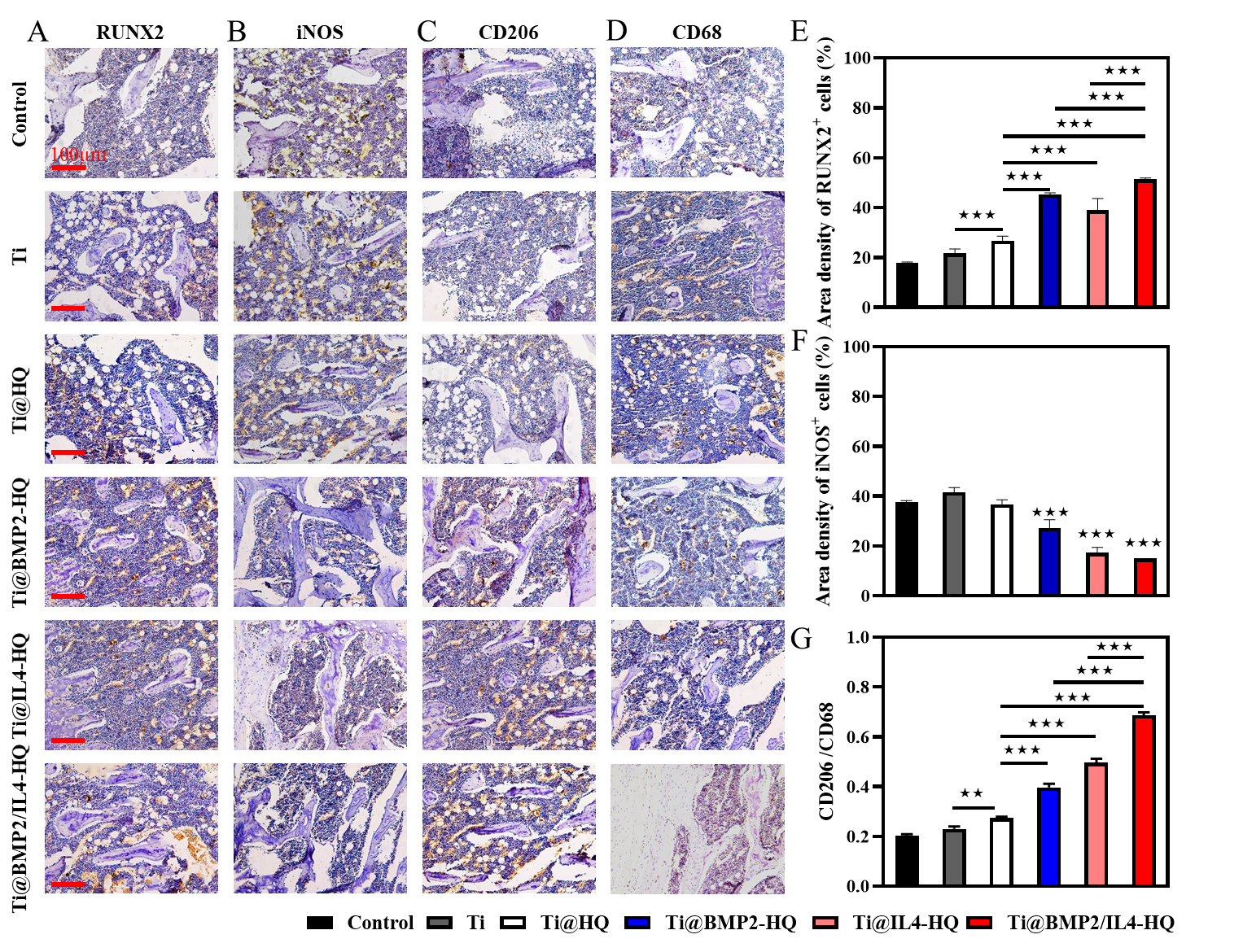


**Figure S14**. Endogenous bone regeneration demonstrated by immunohistochemistry staining. (A-D) Representative immunohistochemistry staining of Runx2 (A), iNOS (B), and CD206 (C), CD68 (D) in defect areas. The Ti@BMP2/IL4-HQ group showed more RUNX2^+^ CD206 cells and increased expression levels in the defect area at 4 weeks. (E) Semi quantification of de novo bone area in (A). (F) Semi quantification of de novo bone area in (B). The statistical significance denoted by *** represents a statistical difference between all other groups. (G) The quantitative analysis of CD206- and CD68-positive areas by the Image-Pro Plus software in sectioned new bone tissues 4 weeks post-operation. Error bars indicate standard deviation (n = 3); **p<0.01 and ***p<0.001.

**Table S1**. Sequences of primers used in the real-time PCR.

| Gene | Forward primer (5’-3’) | Reverse primer (5’-3’) |
| --- | --- | --- |
| COL I | GGCTCCTGCTCCTCTTAG | CAGTTCTTGGTCTCGTCAC |
| ALP | GACCTCCTCGGAAGACACTC | TGAAGGGCTTCTTGTCTGTG |
| OCN | GGTGCAGACCTAGCAGACACCA | AGGTAGCGCCGGAGTCTATTCA |
| RUNX2 | TTACCTACACCCCGCCAGTC | TGCTGGTCTGGAAGGGTCC |

**References**

[1] W. Liu, W. Ou-Yang, C. Zhang, Q. Wang, X. Pan, P. Huang, C. Zhang, Y. Li, D. Kong, W. Wang, Synthetic polymeric antibacterial hydrogel for methicillin-resistant staphylococcus aureus-infected wound healing: nanoantimicrobial self-assembly, drug- and cytokine-free strategy, ACS nano 14(10) (2020) 12905.
